# Objective pulse wave amplitude as a potential systemic correlate in childhood myopia: a digital TCM-based study

**DOI:** 10.3389/fmed.2026.1815599

**Published:** 2026-05-08

**Authors:** Fang Sha, Bin Guo, Dongfang Wang, Yuanyuan Hu, Yane Gao, Xiujuan Du, Meihua Ding, Jiaojiao Feng, Jike Song, Hongsheng Bi

**Affiliations:** 1 Shandong University of Traditional Chinese Medicine, Jinan, China; 2 Affiliated Eye Hospital of Shandong University of Traditional Chinese Medicine, Jinan, China; 3 Shandong Provincial Key Laboratory of Integrated Traditional Chinese and Western Medicine for Prevention and Therapy of Ocular Diseases, Shandong Academy of Eye Disease Prevention and Therapy, Jinan, China; 4 Shandong Provincial Clinical Medical Research Center of Optometry and Children Visual Impairment Prevention and Control, Jinan, China

**Keywords:** children, digital Traditional Chinese Medicine, integrated diagnosis, myopia, objective pulse diagnosis

## Abstract

**Background:**

Childhood myopia is increasingly recognized as a condition with systemic underpinnings, aligning with the holistic perspective of Traditional Chinese Medicine (TCM). This study aimed to explore associations between childhood myopia and objectively measured TCM pulse, tongue, and face diagnostic parameters using digital instruments.

**Methods:**

In this cross-sectional, school-based study, 873 children aged 7–14 years from Shandong Province, China, underwent comprehensive examinations. Myopia was defined as a cycloplegic spherical equivalent (SE) ≤ −0.50 D. Objective parameters of pulse (using the DS01-DI pulse diagnosis instrument), tongue, and face (using the DS01-B system) were automatically acquired. Associations with myopia, SE, and axial length (AL) were evaluated using multivariable logistic regression and generalized linear models, adjusting for age, sex, BMI, parental myopia, time spent on near work and outdoor activities, and TCM body constitution.

**Results:**

Among 873 children, 464 (53.15%) had myopia. After covariate adjustment, the amplitude of the main pulse wave (h1) was independently and negatively associated with myopia in all children (Model 1: OR = 0.834, *P* < 0.001; Model 2: OR = 0.831, *P* < 0.001). This association remained significant in both age subgroups (7–10 and 11–14 years). Higher h1 correlated with greater SE and shorter AL in the older group. No significant associations were found between myopia and any quantified tongue or facial parameters. Myopic children also showed a significantly higher prevalence of biased TCM constitutions compared to those with a balanced constitution.

**Conclusion:**

Reduced h1 amplitude is a systemic correlate of childhood myopia, whereas tongue and facial features showed no significant associations. These findings support the potential of pulse-based systemic assessment in integrated myopia research.

## 1 Introduction

Myopia has become a global public health crisis, with projections that half the world’s population may be affected by 2050 (1). In East Asia, the prevalence among children and adolescents is particularly alarming, exceeding 80% in some regions (2–4). Beyond its high prevalence, myopia—especially high myopia-carries significant risks of vision-threatening complications such as retinal detachment, myopic maculopathy, and glaucoma, imposing a substantial socioeconomic burden (5, 6). Notably, a recent large-scale cohort study from China reported a 28.2% increase in myopia prevalence over 4 years among school-aged children, underscoring the rapid progression of myopia in this population (7).

The pathogenesis of myopia is multifactorial, involving a complex interplay of genetic predisposition and environmental exposures such as prolonged near work and limited outdoor time (8–12). Contemporary research has predominantly focused on localized ocular mechanisms, including scleral remodeling, retinal signaling, and choroidal perfusion (13–15). However, growing evidence suggests that myopia is also associated with systemic physiological parameters such as body height, pre-hypertension, and glucose metabolism (16–18). This indicates that myopia may not be an isolated ocular condition but rather a disorder with systemic correlates—a perspective that aligns with the holistic framework of Traditional Chinese Medicine (TCM).

In TCM theory, visual function is intimately connected to the state of the *zang-fu organs*, *Qi*, and *blood*. The eyes are considered the outward manifestation of the *Liver*, and their nourishment depends on the adequacy of *Qi* and *blood*. Myopia is traditionally attributed to patterns such as “*Liver and Blood deficiency*,” “*Qi deficiency*,” or “*Congenital endowment deficiency*,” reflecting an intrinsic systemic dimension to its etiology (19). TCM diagnostic methods—particularly pulse, tongue, and face diagnosis—offer a unique window into these systemic imbalances. However, their clinical application has historically relied on subjective interpretation, limiting reproducibility and integration with modern evidence-based medicine (20, 21).

The advent of digital and intelligent TCM technologies provides an opportunity to objectify and quantify these traditional diagnostic modalities (22). Digital pulse waveform analysis has been successfully applied to characterize hypertension, coronary heart disease, and other systemic conditions (23–26). Computerized tongue and facial image analysis have shown promise in screening for metabolic disorders, cancers, and other chronic diseases (27–34). Meanwhile, artificial intelligence has been successfully applied to myopia, achieving high accuracy in risk classification and pathologic myopia detection using fundus images (35, 36). However, these digital approaches primarily rely on ocular imaging rather than systemic physiological parameters.

Despite this progress, the application of digital TCM diagnostics to childhood myopia remains unexplored. It is unknown whether objectively measured pulse, tongue, or facial parameters reflect the systemic aspects of myopia pathogenesis as posited by TCM theory. Addressing this gap is critical for advancing an integrated understanding of myopia—one that connects localized refractive characteristics with whole-body physiology. Such an approach could inform more comprehensive strategies for myopia prevention and management.

Therefore, this population-based, cross-sectional study aimed to investigate the associations between childhood myopia and objectively quantified parameters derived from digital pulse, tongue, and face diagnoses. By leveraging digital TCM instruments, we sought to provide empirical, quantitative evidence that links systemic diagnostic markers with ocular refractive status.

## 2 Materials and methods

### 2.1 Participants

This cross-sectional, school-based study was conducted in Huantai County, Shandong Province, China, from September to October 2023. Participants were children aged 7–14 years. The study protocol was approved by the Ethics Committee of the Affiliated Eye Hospital of Shandong University of Traditional Chinese Medicine (No. HEC-KS-2022025KY). Written informed consent was obtained from all participants and their guardians in accordance with the Declaration of Helsinki.

Sample size was calculated using OpenEpi,^1^ with a significance level of 0.05 (37). Based on reported myopia prevalence rates of 54.00% in primary students and 78.18% in middle school students (38), and a margin of error of 5%, the minimum sample sizes were estimated as 382 for primary school students and 263 for middle school students. These numbers were inflated by a design effect of 1.2 to account for stratified random sampling and potential data loss, resulting in a target sample of 774 children.

Using stratified random sampling, 911 children were recruited from four schools (two primary and two middle schools). From each primary school grade (five grades per school), three classes were randomly selected, and 20 children were randomly chosen per class. From each middle school grade (four grades per school), two classes were randomly selected, with 20 children randomly sampled per class.

### 2.2 Inclusion and exclusion criteria

Inclusion criteria were: (1) age 7–14 years; (2) best corrected visual acuity ≥ 20/20; (3) complete examination data; and (4) signed informed consent.

Exclusion criteria were: (1) failure to meet inclusion criteria; (2) history of orthokeratology lens wear; (3) ocular diseases such as strabismus, amblyopia, fundus diseases, or ocular injury; (4) systemic diseases including congenital cardiovascular diseases or acute illnesses (e.g., fever, diarrhea); and (5) uncooperative behavior during examinations.

### 2.3 General and ophthalmic examinations

A standardized questionnaire adapted from the Refractive Error Study in Children (RESC) was administered to collect demographic information, parental myopia history, and lifestyle factors (indoor near work and outdoor activity time on weekdays and weekends).

Prior to refractive assessment, two ophthalmologists performed slit-lamp and fundus examinations to exclude pre-existing ocular pathology. The other examinations were performed by trained optometrists. Visual acuity was measured at 3 m using a “E” chart (#600722, Good-Lite Co., Elgin, IL, United States). Non-contact tonometry (Topcon CT80) and autorefraction (Nidek ARK-1) were performed before and after cycloplegia. Cycloplegia was induced using 1% cyclopentolate hydrochloride (Alcon, United States) administered three times at 5-min intervals. After 30 min of eye closure, pupillary light reflex and diameter were assessed. Cycloplegic autorefraction was performed only if the pupillary light reflex was absent or pupil diameter exceeded 6 mm. Axial length (AL) was measured three times using laser interferometry (IOL-Master 500, Carl Zeiss), and the average value was recorded. Height and weight were measured to calculate body mass index (BMI; kg/m^2^).

Spherical equivalent (SE) was calculated as sphere+ 1/2 cylinder. Myopia was defined as cycloplegic SE ≤ −0.50 D per International Myopia Institute criteria (39). Data from the right eye were analyzed.

### 2.4 Collection of digital TCM diagnosis data

All digital diagnostic procedures (pulse, tongue, and facial examinations) were performed by trained TCM practitioners. All measurements were conducted from 9:00 a.m. to 11:00 a.m.

**FIGURE 1:**
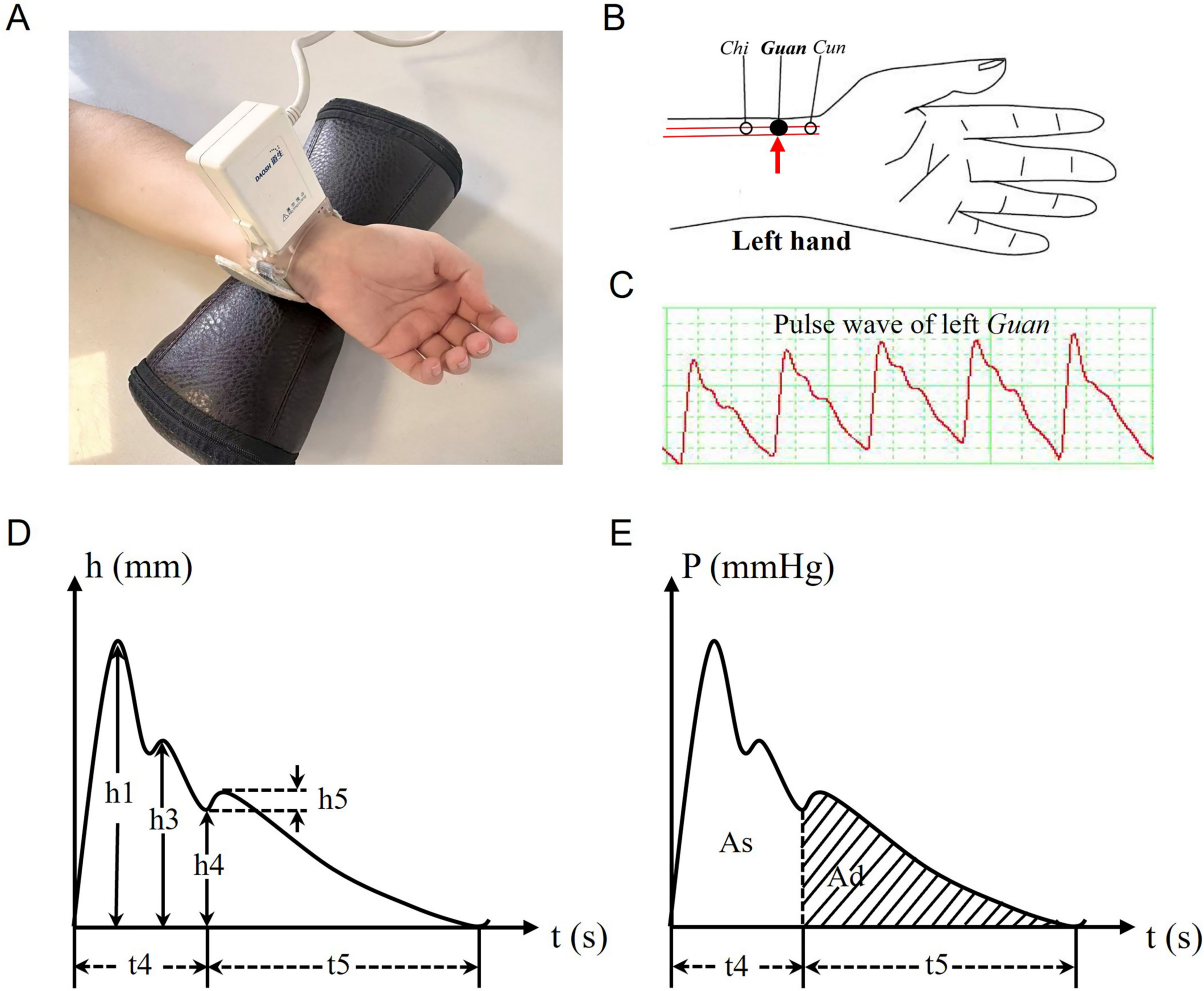
The DS01-DI model pulse diagnosis instrument and the basic structure of pulse wave. **(A)** DS01-DI model pulse diagnosis instrument and data collection of pulse diagnosis at the left wrist. **(B)** The *Cun*, *Guan*, and *Chi* positions on the left wrist. Pulse wave acquisition at the left *Guan* position (red arrow) in the study. **(C)** Output of the pulse wave at the left *Guan* position. **(D,E)** The basic structure of pulse wave.

**FIGURE 2:**
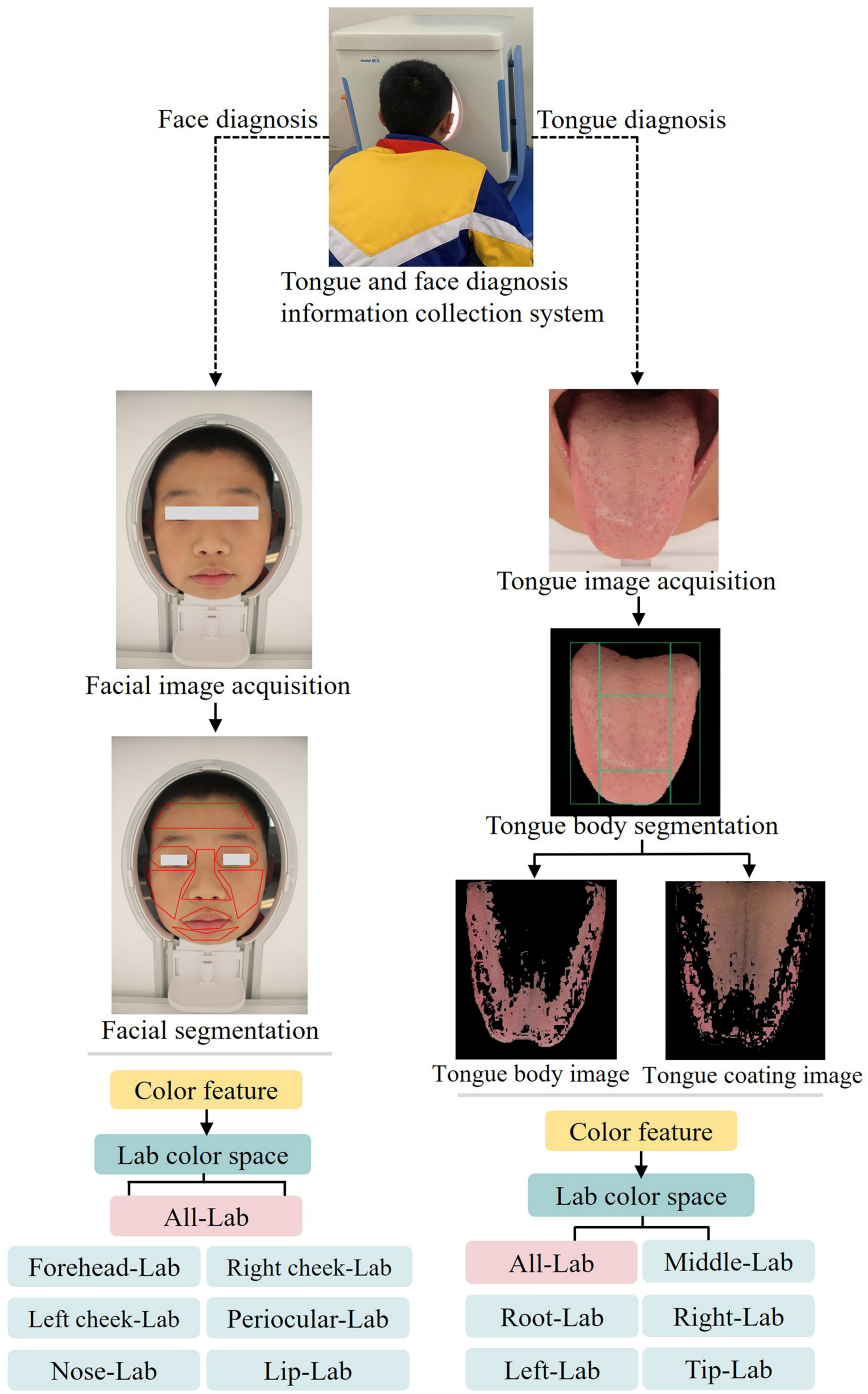
The process of extracting the color features of the tongue and face images.

(1) Pulse diagnosis: The DS01-DI pulse diagnosis instrument (Shanghai Daosheng Medical Technology Co., Ltd.; Registration No. 20172270556), whose reliability and validity for pulse waveform analysis have been demonstrated in previous studies (40, 41), was used to acquire radial artery pulse waves (Figure 1A). After resting for at least 10 min, participants sat with the left forearm supported at heart level, palm upward. The pressure sensor was placed at the left *Guan* position (Figure 1B), which corresponds to the Liver in TCM theory and is located ventral to the radial styloid process (42). The device automatically adjusted contact pressure to optimize signal acquisition. A stable 30-s tracing was recorded, and the resulting pulse wave output is shown in Figure 1C. Dedicated software extracted time-domain features from the pulse wave structure (Figures 1D,E), including seven amplitude parameters (h1, h3, h4, h5, h3/h1, h4/h1, h5/h1), three time parameters (t4, t5, t), and two area parameters (As, Ad). The definitions of these features are provided in Supplementary Table 1.

(2) Tongue and face diagnoses: The DS01-B tongue and face diagnosis information collection system (Shanghai Daosheng; Registration No. 20202200062), whose credibility for color feature extraction has been supported by previous studies (43, 44), was used under constant, simulated natural lighting (Figure 2). For tongue image acquisition, participants placed their chin on a rest, extended the tongue fully with the tip down, and images were captured promptly. Mouth rinsing was performed if necessary to remove food residue. For facial images, participants sat with a neutral expression, removed glasses, and adjusted hair to fully expose the face. The system automatically segmented the tongue image into five regions (middle, root, right margin, left margin, tip) and the facial image into seven regions (forehead, right cheek, left cheek, periocular area, nose, lip; chin excluded). Figure 2 illustrates the process of extracting color features from the tongue and face images. Features were quantified in the CIELAB color space, with L (lightness), a (red-green axis), and b (yellow-blue axis) values extracted for analysis. Tongue shape was classified as medium, swollen, or thin.

### 2.5 Assessment of TCM body constitution

Each body constitution type was assessed using the validated “7–14 years old questionnaire of constitution in TCM” (45). For younger children (ages 7–8), parents assisted in completion; older children completed it with the help of TCM-trained investigators. The questionnaire was divided into nine subscales, one for each body constitution type and consisting of 5–7 questions, for a total of 51 questions. Each question was scored on a 5-point frequency and intensity scale and related to a specific body constitution: balanced constitution (6 questions), Qi deficiency (7), Yang deficiency (6), Yin deficiency (5), phlegm dampness (7), damp-heat (5), blood-stasis (6), Qi stagnation (7), and special constitution (6). For each body constitution, the raw score was obtained by summing the response numbers for the related questions. This raw score was then converted to a 0–100 scale using the formula (46): Converted score = [(raw score-N)/N] × 25, where N represents the number of questions. Participants were classified using the following criteria: (1) Balanced constitution: a balanced score ≥ 60 concurrently with all eight biased constitution scores < 40. (2) Biased constitution: if the score for any biased constitution was ≥ 40. In cases where multiple biased constitutions scored ≥ 40, the one with the highest score was designated as the primary type.

### 2.6 Statistical analysis

Statistical analyses were performed using SPSS 24.0 (Inc., Chicago, IL, United States). Continuous variables are presented as mean ± standard deviation (SD) (normal distribution) or median with interquartile range (non-normal). Categorical variables were presented as n (%). Group comparisons used independent *t*-tests, Mann-Whitney U tests, Chi-square tests, or Fisher’s exact test as appropriate. Trends across age groups were assessed using polynomial linear correlation. Bivariate relationships were evaluated using Pearson’s or Spearman’s correlation. To address multiple comparisons, the Benjamini-Hochberg procedure was applied to control the false discovery rate (FDR). Multicollinearity was assessed using variance inflation factor (VIF). Variables with corrected *P* < 0.10 in univariate analysis and VIF < 5 were included in multivariable models. Logistic regression was used to assess associations between TCM diagnostic parameters and myopia, expressed as odds ratio (OR) with a 95% confidence interval (CI). Generalized linear model (GLM) evaluated associations with SE and AL, expressed as coefficients (β) and 95% CI. Model 1 and Model 2 were both fully adjusted for covariates (age, sex, BMI, parental myopia, indoor near work time per day on weekdays/weekends, outdoor activity time per day on weekdays/weekends, and TCM body constitution), but differed in that they incorporated global versus local region parameters of tongue and facial images, respectively. Receiver operating characteristic (ROC) curve analysis with the Youden index was used to determine parameter thresholds. All tests were two-sided, with *P* < 0.05 considered statistically significant.

## 3 Results

### 3.1 Study population characteristics

A total of 911 children were initially enrolled. After exclusions, 38 children were excluded due to amblyopia (*n* = 2), history of orthokeratology lens wear (*n* = 4), or incomplete examination data (*n* = 32). The final analysis included 873 children (95.83% of the recruited sample), with a mean age of 10.06 ± 2.19 years (range 7–14). Among them, 449 (51.43%) were female, and 464 (53.15%) had myopia. Demographic and clinical characteristics of the participants are summarized in Table 1.

**TABLE 1:** Demographic characteristics between myopic and non-myopic children.

Variables	Total (*n* = 873)	Myopia (*n* = 464)	Non-myopia (*n* = 409)	*P* value	*P*-FDR
Age (years)	10.06 ± 2.19	10.66 ± 2.16	9.38 ± 2.01	<0.001^a^	<0.001
Sex, n (%)					
Male	424 (48.57)	226 (48.71)	198 (48.41)	0.930^b^	0.930
Female	449 (51.43)	238 (51.29)	211 (51.59)		
BMI (kg/m^2^)	18.85 ± 3.77	19.14 ± 3.73	18.53 ± 3.80	0.017^a^	0.063
Number of myopic parents, n (%)					
0	298 (34.14)	140 (30.17)	158 (38.63)	0.030^b^	0.081
1	326 (37.34)	182 (39.22)	144 (35.21)		
2	249 (28.52)	142 (30.60)	107 (26.16)		
Indoor near work time per day on weekdays (hours), n (%)					
<4	282 (32.30)	130 (28.02)	152 (37.16)	0.001^b^	0.007
≥ 4 to<8	440 (50.40)	237 (51.08)	203 (49.63)		
≥8	151 (17.30)	97 (20.91)	54 (13.20)		
Indoor near work time per day on weekends (hours), n (%)					
<4	356 (40.78)	175 (37.72)	181 (44.25)	0.094^b^	0.179
≥ 4 to<8	398 (45.59)	218 (46.98)	180 (44.01)		
≥8	119 (13.63)	71 (15.30)	48 (11.74)		
Outdoor activity time per day on weekdays (hours), n (%)					
<1	430 (49.26)	259 (55.82)	171 (41.81)	<0.001^b^	<0.001
≥ 1 to<2	323 (37.00)	157 (33.84)	166 (40.59)		
≥2	120 (13.75)	48 (10.34)	72 (17.60)		
Outdoor activity time per day on weekends (hours), n (%)					
<1	270 (30.93)	169 (36.50)	101 (24.69)	<0.001^b^	0.004
≥ 1 to<2	321 (36.77)	163 (35.21)	158 (38.63)		
≥2	281 (32.19)	131 (28.29)	150 (36.67)		
Body constitution type, n (%)					
Balance	446(51.09)	203(45.52)	243(54.48)	0.001^c^	0.007
Qi stagnation	171(19.59)	108(63.16)	63(36.84)		
Yin deficiency	117(13.40)	72(61.54)	45(38.46)		
Yang deficiency	61(6.99)	37(60.66)	24(39.34)		
Qi deficiency	37(4.24)	20(54.05)	17(45.95)		
Special constitution	22(2.52)	14(63.64)	8(36.36)		
Damp-heat	8(0.92)	3(37.50)	5(62.50)		
Phlegm dampness	6(0.69)	3(50.00)	3(50.00)		
Blood-stasis	5(0.57)	4(80.00)	1(20.00)		

^a^Independent-Samples *t*-test was used in continuous normally distributed variable comparisons.

^b^Chi-square test was used in classification variable comparisons.

^c^Fisher’s exact test was used in classification variable comparisons. BMI, body mass index; FDR, false discovery rate.

The distribution of TCM body constitution types is shown in Table 1. *Balanced constitution* was most prevalent (446/873, 51.09%), followed by *Qi stagnation* (171/873, 19.59%), *Yin deficiency* (117/873, 13.40%), *Yang deficiency* (61/873, 6.99%), and *Qi deficiency* (37/873, 4.24%). The distribution differed markedly between myopic and non-myopic children. Myopic children had a significantly lower prevalence of the balanced constitution and higher prevalence of biased constitutions. Accordingly, myopia prevalence varied significantly across constitution types (χ^2^ = 25.246, *P* = 0.001), being lowest in the balanced group (203/446, 45.52%) and exceeding 60% in those with *Qi stagnation* (108/171, 63.16%), *Yin deficiency* (72/117, 61.54%), or *Yang deficiency* (37/61, 60.66%).

### 3.2 Comparison of digital TCM parameters between myopic and non-myopic children

Univariate comparisons are presented in Table 2. Regarding pulse wave features, the myopic group showed significantly lower amplitudes of the main wave (h1), the front wave of dicrotic pulse (h3), and the dicrotic notch (h4) (all *P* < 0.05).

**TABLE 2:** Comparison of pulse, tongue, and face diagnostic parameters.

Variables	Total (*n* = 873)	Myopia (*n* = 464)	Non-myopia (*n* = 409)	*P*-value	*P*-FDR
Pulse conditiona					
h1 (mm)	13.45 ± 3.96	13.17 ± 3.73	13.76 ± 4.19	0.029	0.081
h3 (mm)	10.16 ± 2.78	9.98 ± 2.68	10.37 ± 2.88	0.041	0.099
h4 (mm)	6.28 ± 1.99	6.15 ± 1.92	6.43 ± 2.05	0.038	0.096
h5 (mm)	0.73 ± 0.50	0.72 ± 0.50	0.74 ± 0.50	0.526	0.651
t4 (s)	0.30 ± 0.05	0.30 ± 0.06	0.29 ± 0.05	0.573	0.688
t5 (s)	0.39 ± 0.11	0.39 ± 0.11	0.39 ± 0.10	0.380	0.529
t (s)	0.68 ± 0.13	0.69 ± 0.13	0.68 ± 0.13	0.480	0.614
h3/h1	0.78 ± 0.19	0.79 ± 0.21	0.78 ± 0.17	0.414	0.567
h4/h1	0.48 ± 0.15	0.49 ± 0.16	0.48 ± 0.13	0.544	0.663
h5/h1	0.06 ± 0.04	0.06 ± 0.04	0.06 ± 0.04	0.471	0.612
As	61.38 ± 21.23	60.56 ± 19.57	62.31 ± 22.96	0.225	0.344
Ad	32.35 ± 14.72	32.03 ± 14.65	32.71 ± 14.81	0.497	0.625
Tongue colora					
All-L	53.39 ± 3.06	53.23 ± 3.14	53.57 ± 2.98	0.103	0.187
All-a	20.03 ± 1.96	20.00 ± 2.03	20.06 ± 1.89	0.626	0.688
All-b	10.08 ± 1.66	10.64 ± 1.72	10.99 ± 1.56	0.002	0.011
Middle-L	56.52 ± 3.57	56.34 ± 3.63	56.73 ± 3.50	0.106	0.184
Middle-a	19.40 ± 2.66	19.44 ± 2.66	19.34 ± 2.66	0.574	0.678
Middle-b	10.05 ± 1.90	9.91 ± 1.98	10.22 ± 1.78	0.015	0.059
Root-L	49.25 ± 5.10	49.19 ± 5.10	49.32 ± 5.11	0.705	0.753
Root-a	16.12 ± 2.46	16.06 ± 2.46	16.20 ± 2.46	0.376	0.533
Root-b	10.12 ± 1.96	9.96 ± 2.04	10.31 ± 1.85	0.008	0.035
Right-L	52.91 ± 3.12	52.76 ± 3.15	53.09 ± 3.08	0.121	0.205
Right-a	20.16 ± 1.96	20.14 ± 2.03	20.19 ± 1.88	0.684	0.741
Right-b	10.96 ± 1.68	10.81 ± 1.74	11.14 ± 1.59	0.005	0.024
Left-L	52.83 ± 3.30	52.68 ± 3.26	53.01 ± 3.34	0.140	0.232
Left-a	20.20 ± 1.99	20.18 ± 2.05	20.22 ± 1.93	0.746	0.776
Left-b	11.01 ± 1.70	10.83 ± 1.76	11.21 ± 1.62	0.001	0.007
Tip-L	55.62 ± 3.11	55.45 ± 3.22	55.80 ± 2.96	0.094	0.179
Tip-a	23.41 ± 2.76	23.34 ± 2.81	23.49 ± 2.71	0.426	0.573
Tip-b	11.36 ± 1.78	11.23 ± 1.81	11.50 ± 1.74	0.026	0.078
Tongue shapec					
Medium	513 (58.76)	269 (57.97)	244 (59.66)	0.580	0.675
Swollen	253 (28.98)	141 (30.39)	112 (27.38)		
Thin	107 (12.26)	54 (11.64)	53 (12.96)		
Tongue coating colorb					
All-L	18.08 (8.75, 30.37)	16.83 (8.32, 29.15)	19.66 (9.34, 31.52)	0.085	0.179
All-a	5.61 (2.90, 9.70)	5.43 (2.75, 9.36)	6.13 (3.18, 9.99)	0.086	0.177
All-b	3.56 (1.73, 6.45)	3.34 (1.64, 6.18)	3.85 (1.89, 6.80)	0.035	0.091
Middle-L	16.26 (4.48, 38.76)	14.92 (4.19, 38.02)	18.20 (4.63, 40.25)	0.268	0.402
Middle-a	5.08 (1.63, 11.25)	4.88 (1.59, 11.00)	5.57 (1.72, 11.66)	0.371	0.536
Middle-b	2.87 (1.01, 6.87)	2.49 (0.97, 6.72)	3.21 (1.05, 7.18)	0.147	0.239
Root-L	36.26 (15.69, 48.34)	34.55 (13.91, 47.73)	38.06 (17.55, 49.04)	0.104	0.184
Root-a	10.60 (4.87, 13.74)	10.12 (4.65, 13.33)	11.20 (5.57, 14.08)	0.020	0.068
Root-b	7.09 (2.84, 10.57)	6.87 (2.53, 10.28)	7.76 (3.19, 10.76)	0.045	0.106
Right-L	16.51 (6.55, 31.30)	15.50 (6.22, 30.27)	18.11 (7.47, 32.49)	0.093	0.181
Right-a	5.44 (2.28, 10.18)	5.11 (2.15, 10.05)	5.67 (2.61, 10.41)	0.091	0.182
Right-b	3.30 (1.38, 6.69)	3.10 (1.27, 6.50)	3.69 (1.49, 6.90)	0.047	0.108
Left-L	16.33 (6.19, 31.73)	15.16 (5.67, 29.82)	18.68 (7.44, 33.20)	0.023	0.072
Left-a	5.54 (2.27, 10.32)	5.17 (2.01, 9.66)	6.17 (2.63, 10.83)	0.017	0.063
Left-b	3.48 (1.33, 6.72)	3.23 (1.20, 6.19)	3.88 (1.61, 7.10)	0.010	0.041
Tip-L	4.54 (1.54, 12.79)	4.62 (1.46, 14.14)	4.39 (1.57, 11.17)	0.467	0.617
Tip-a	1.72 (0.74, 4.77)	1.77 (0.74, 5.04)	1.64 (0.75, 4.20)	0.318	0.468
Tip-b	1.02 (0.57, 2.36)	1.03 (0.56, 2.46)	0.99 (0.57, 2.17)	0.619	0.700
Facial colora					
All-L	56.07 ± 2.20	55.83 ± 2.11	56.34 ± 2.27	0.001	0.007
All-a	15.05 ± 1.34	15.03 ± 1.36	15.06 ± 1.32	0.732	0.772
All-b	18.15 ± 1.60	17.93 ± 1.65	18.41 ± 1.50	<0.001	<0.001
Forehead-L	55.24 ± 3.70	54.92 ± 3.66	55.61 ± 3.71	0.005	0.024
Forehead-a	15.73 ± 1.69	15.63 ± 1.71	15.84 ± 1.67	0.065	0.141
Forehead-b	15.71 ± 1.69	15.61 ± 1.70	15.82 ± 1.68	0.059	0.132
Right cheek-L	62.09 ± 2.33	61.92 ± 2.16	62.27 ± 2.49	0.029	0.084
Right cheek-a	15.29 ± 1.78	15.28 ± 1.79	15.31 ± 1.77	0.842	0.864
Right cheek-b	20.14 ± 2.35	19.78 ± 2.43	20.55 ± 2.18	<0.001	<0.001
Left cheek-L	60.36 ± 2.63	60.25 ± 2.55	60.47 ± 2.72	0.217	0.339
Left cheek-a	15.04 ± 1.79	15.07 ± 1.80	15.00 ± 1.78	0.619	0.700
Left cheek-b	19.44 ± 2.30	19.08 ± 2.39	19.85 ± 2.11	<0.001	<0.001
Periocular-L	49.87 ± 2.73	49.67 ± 2.84	50.10 ± 2.60	0.020	0.068
Periocular-a	13.36 ± 1.21	13.31 ± 1.22	13.42 ± 1.21	0.168	0.267
Periocular-b	16.64 ± 1.75	16.37 ± 1.80	16.94 ± 1.64	<0.001	<0.001
Nose-L	60.64 ± 2.20	60.44 ± 2.11	60.88 ± 2.28	0.003	0.016
Nose-a	14.72 ± 1.45	14.70 ± 1.48	14.75 ± 1.42	0.615	0.705
Nose-b	20.80 ± 2.11	20.50 ± 2.15	21.13 ± 2.02	<0.001	<0.001
Lip-L	52.13 ± 2.87	51.76 ± 2.75	52.54 ± 2.94	<0.001	<0.001
Lip-a	18.93 ± 1.70	18.94 ± 1.67	18.92 ± 1.75	0.879	0.890
Lip-b	16.78 ± 1.62	16.58 ± 1.65	17.01 ± 1.57	<0.001	<0.001

Data are expressed as mean ± SD for normally distributed variables, or as medium and interquartile range for non-normally distributed variables.

*^a^*Independent *t*-test was used in continuous normally distributed variable comparisons.

*^b^*Mann-Whitney U test was used in continuous non-normally distributed variable comparisons.

*^c^*Chi-square test was used in classification variable comparisons. The color variables (L, a, b) were defined in terms of the CIELAB space, in which L (lightness), a (red-green axis), b (Yellow-blue axis). FDR, false discovery rate.

For tongue and facial color parameters, several significant differences were observed. Myopic children had significantly lower L (lightness) values for the left lateral region of the tongue coating, as well as for the overall face and specific facial regions (forehead, right cheek, periocular area, nose, lip) (all *P* < 0.05). The a (red-green) values were lower in the root and left lateral regions of the tongue coating (all *P* < 0.05). Significant reductions in b (yellow-blue) values were also widespread in the myopic group, affecting almost all measured regions of the tongue body, tongue coating, and face (all *P* < 0.05). No significant differences in tongue shape were observed.

### 3.3 Association of TCM diagnostic parameters with myopia in multivariable models

The associations between TCM parameters and myopia after adjusting for covariates (age, sex, BMI, parental myopia, indoor near work time per day on weekdays/weekends, outdoor activity time per day on weekdays/weekends, and TCM body constitution) are shown in Table 3. In fully adjusted logistic regression models for all children, only the pulse wave amplitude h1 remained independently and negatively associated with myopia (Model 1: OR = 0.834, 95% CI: 0.784–0.887, *P* < 0.001; Model 2: OR = 0.831, 95% CI: 0.781–0.884, *P* < 0.001). No tongue or facial color/shape parameters showed significant associations.

**TABLE 3:** Multiple logistic regression for pulse, tongue, and face diagnoses and myopia in all children (*n* = 873).

Variables	OR (95% CI)	*P-*value
Model 1a		
h1	0.834 (0.784, 0.887)	<0.001
Age	1.541 (1.393, 1.704)	<0.001
Number of myopic parents		
0	Ref.	
1	1.893 (1.318, 2.718)	0.001
2	2.044 (1.379, 3.030)	<0.001
Indoor near work time per day on weekdays (hours)		
<4	Ref.	
≥ 4 to<8	1.116 (0.791, 1.576)	0.531
≥8	1.639 (1.028, 2.615)	0.038
Outdoor activity time per day on weekdays (hours)		
<1	Ref.	
≥ 1 to<2	0.720 (0.506, 1.023)	0.067
≥2	0.632 (0.402, 0.995)	0.047
Body constitution type, n (%)		
Balance	Ref.	
Qi stagnation	1.983 (1.325, 2.967)	0.001
Yin deficiency	2.339 (1.473, 3.713)	<0.001
Yang deficiency	2.001 (1.070, 3.743)	0.030
Qi deficiency	2.231 (0.991, 5.025)	0.053
Other types^c^	1.396 (0.683, 2.854)	0.360
Model 2b		
h1	0.831 (0.781, 0.884)	<0.001
Age	1.540 (1.391, 1.705)	<0.001
Number of myopic parents		
0	Ref.	
1	1.896 (1.318, 2.728)	0.001
2	2.026 (1.362, 3.013)	<0.001
Indoor near work time per day on weekdays (hours)		
<4	Ref.	
≥ 4 to<8	1.107 (0.784, 1.563)	0.563
≥8	1.602 (1.003, 2.558)	0.049
Outdoor activity time per day on weekdays (hours)		
<1	Ref.	
≥ 1 to<2	0.714 (0.501, 1.018)	0.063
≥2	0.628 (0.399, 0.989)	0.045
Body constitution type, n (%)		
Balance	Ref.	
Qi stagnation	2.001 (1.337, 2.996)	0.001
Yin deficiency	2.327 (1.464, 3.699)	<0.001
Yang deficiency	2.021 (1.072, 3.813)	0.030
Qi deficiency	2.300 (1.021, 5.184)	0.045
Other types^c^	1.480 (0.721, 3.039)	0.286

^a^Model 1 included the global region parameters of tongue and facial images with *P*-FDR < 0.10 in univariate analysis and VIF < 5.

^b^Model 2 included the local region parameters of tongue and facial images with *P*-FDR < 0.10 in univariate analysis and VIF < 5.

^c^Other types include Special constitution, Damp-heat constitution, Phlegm dampness constitution, and Blood-stasis constitution. OR, Odds ratio; CI, Confidence interval.

Age-stratified subgroup analysis (Table 4) provided further insights. In children aged 7–10 years, lower h1 was significantly associated with a higher odds of myopia (OR = 0.894, 95% CI: 0.844–0.947, *P* < 0.001). A similar pattern was observed in the 11–14 years age group, where h1 was negatively associated with myopia in both Model 1 and Model 2 (all *P* < 0.05). Again, no significant associations were found for tongue or face parameters in either age subgroup. Univariate results for age subgroups are provided in Supplementary Table 2.

**TABLE 4:** Age-stratified multiple logistic regression for pulse, tongue, and face diagnoses and myopia.

Variables	OR (95% CI)	*P-*value
7–10 years old (*n* = 557)*		
h1	0.894 (0.844, 0.947)	<0.001
Age	2.131 (1.757, 2.584)	<0.001
Body constitution type, n (%)		
Balance	Ref.	
Qi stagnation	2.358 (1.417, 3.924)	0.001
Yin deficiency	2.664 (1.599, 4.439)	<0.001
Yang deficiency	2.428 (1.209, 5.106)	0.013
Qi deficiency	2.740 (1.113, 6.744)	0.028
Other types^c^	1.233 (0.425, 3.579)	0.700
**11–14 years old (*n* = 316)**		
Model 1a		
h1	0.847 (0.765, 0.939)	0.002
Age	1.509 (1.167, 1.952)	0.002
Outdoor activity time per day on weekdays (hours)		
<1	Ref.	
≥ 1 to<2	0.698 (0.389, 1.251)	0.227
≥ 2	0.423 (0.191, 0.933)	0.033
Outdoor activity time per day on weekends (hours)		
<1	Ref.	
≥ 1 to<2	0.685 (0.367, 1.280)	0.236
≥ 2	0.422 (0.211, 0.847)	0.015
Model 2b		
h1	0.846 (0.762, 0.938)	0.002
Age	1.484 (1.137, 1.937)	0.004
Outdoor activity time per day on weekdays (hours)		
<1	Ref.	
≥ 1 to<2	0.652 (0.359, 1.184)	0.160
≥ 2	0.426 (0.191, 0.950)	0.037
Outdoor activity time per day on weekends (hours)		
<1	Ref.	
≥ 1 to<2	0.691 (0.369, 1.296)	0.249
≥ 2	0.414 (0.205, 0.835)	0.014

*For children aged 7–10 years, univariate analysis showed no significant association of tongue or face diagnosis parameters with myopia; therefore, only pulse diagnosis parameters with *P*-FDR < 0.10 in univariate analysis and VIF < 5 were included in the multivariate model.

*^a^*Model 1 included the global region parameters of tongue and facial images with *P*-FDR < 0.10 in univariate analysis and VIF < 5.

*^b^*Model 2 included the local region parameters of tongue and facial images with *P*-FDR < 0.10 in univariate analysis and VIF < 5.

*^c^*Other types include Special constitution, Damp-heat constitution, Phlegm dampness constitution, and Blood-stasis constitution. OR, Odds ratio; CI, Confidence interval.

### 3.4 Age-stratified trends, thresholds, and correlations with refractive parameters

**FIGURE 3:**
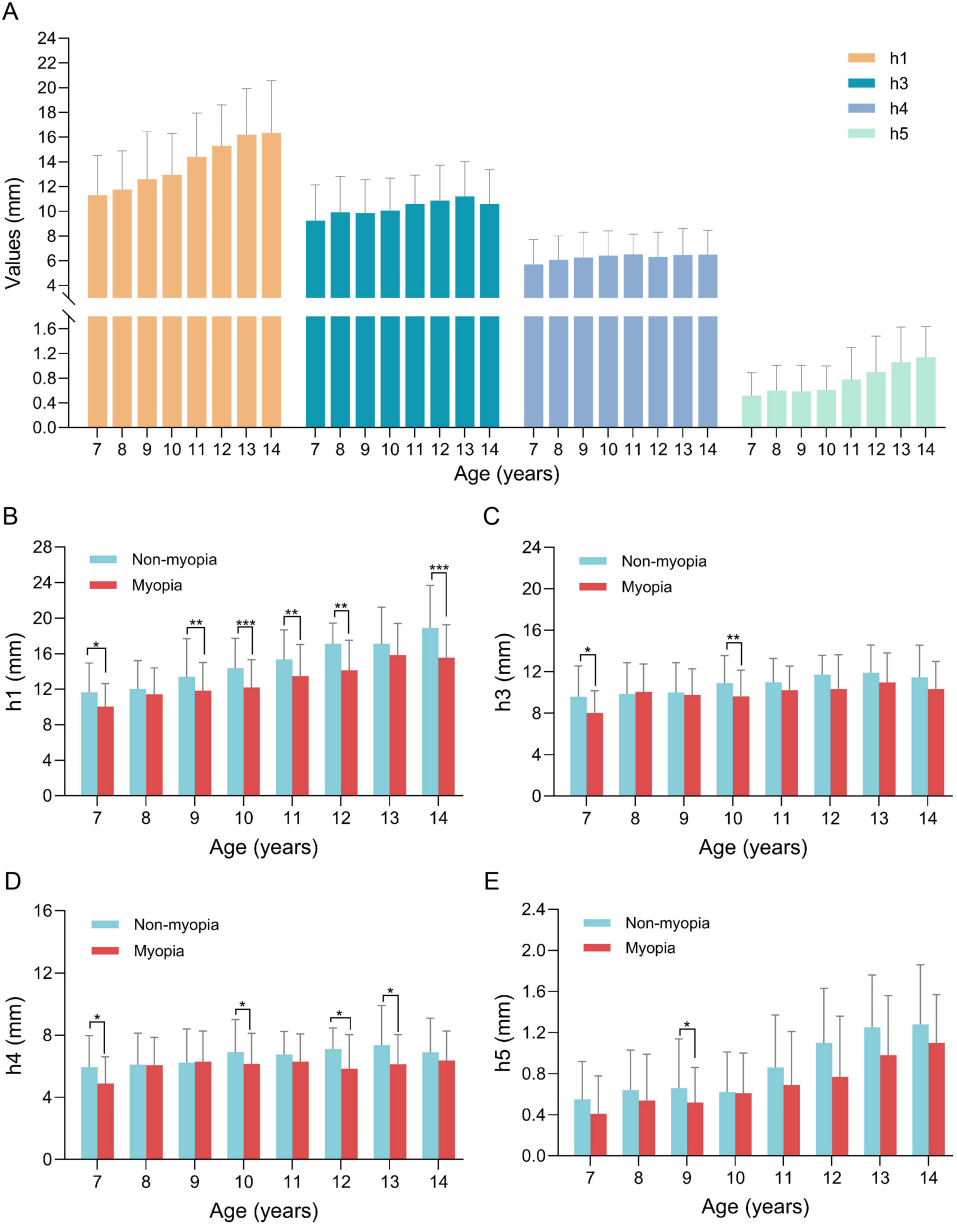
Pulse wave amplitudes stratified by age and refractive state. **(A)** Values of pulse wave amplitudes (h1, h3, h4, h5) stratified by age. **(B)** The amplitude of h1 stratified by age and refractive state. **(C)** The amplitude of h3 stratified by age and refractive state. **(D)** The amplitude of h4 stratified by age and refractive state. **(E)** The amplitude of h5 stratified by age and refractive state. *P < 0.05, **P < 0.01, and ***P < 0.001.

Figure 3 illustrates the trends of pulse wave amplitudes (h1, h3, h4, h5) across age groups. All four amplitudes increased significantly with age in the total population and in both myopic and non-myopic children separately (all *P* for trend < 0.05). Notably, within specific age brackets, the myopic group consistently showed lower h1 values than the non-myopic group (Figure 3B).

To explore the potential clinical utility of h1 as an age-specific screening indicator, we performed ROC curve analysis to determine cutoffs for distinguishing myopic from non-myopic children in each age group (Table 5). The h1 threshold increased progressively with age, from 9.884 mm at 7 years to 15.417 mm at 14 years, indicating that a single fixed cutoff is inappropriate. However, for children aged 8 and 13 years, the AUC was not statistically significant (*P* > 0.05), and no reliable threshold could be established.

**TABLE 5:** Age-specific ROC-derived thresholds of h1 for myopia classification.

Age	*n*	AUC (95% CI)	Sensitivity	Specificity	*P-*value	Threshold (mm)
7	99	0.667(0.532, 0.803)	0.617	0.782	0.019	9.884
8	142	0.548(0.452, 0.644)	NA	NA	0.331	NA
9	170	0.610(0.525, 0.695)	0.698	0.660	0.013	12.786
10	146	0.688(0.597, 0.778)	0.611	0.765	<0.001	12.861
11	101	0.628(0.519, 0.737)	0.627	0.680	0.027	14.371
12	44	0.741(0.592, 0.889)	0.741	0.765	0.008	15.083
13	66	0.568(0.410, 0.726)	NA	NA	0.396	NA
14	105	0.718(0.599, 0.836)	0.638	0.680	0.001	15.417

AUC, Area Under the Curve; ROC, Receiver Operating Characteristic; NA, not applicable, indicating that the AUC was not statistically significant (*P* > 0.05) and no reliable cutoff could be determined.

**FIGURE 4:**
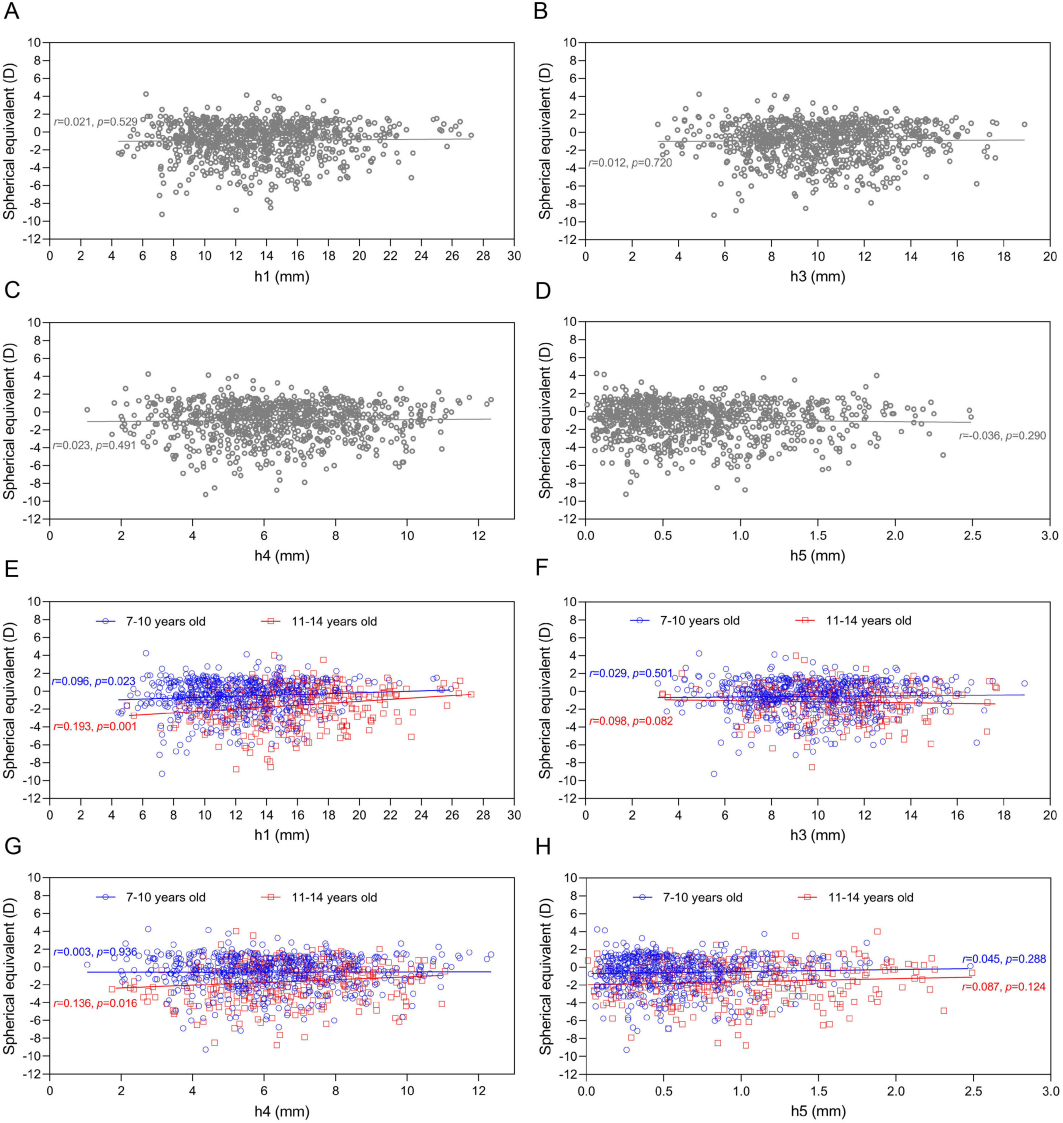
Association between spherical equivalent (SE) and pulse wave amplitude. **(A–D)** Association between SE and h1, h3, h4, and h5 in all children. **(E–H)** Association between SE and h1, h3, h4, and h5 in children aged 7–10 years and 11–14 years.

**FIGURE 5:**
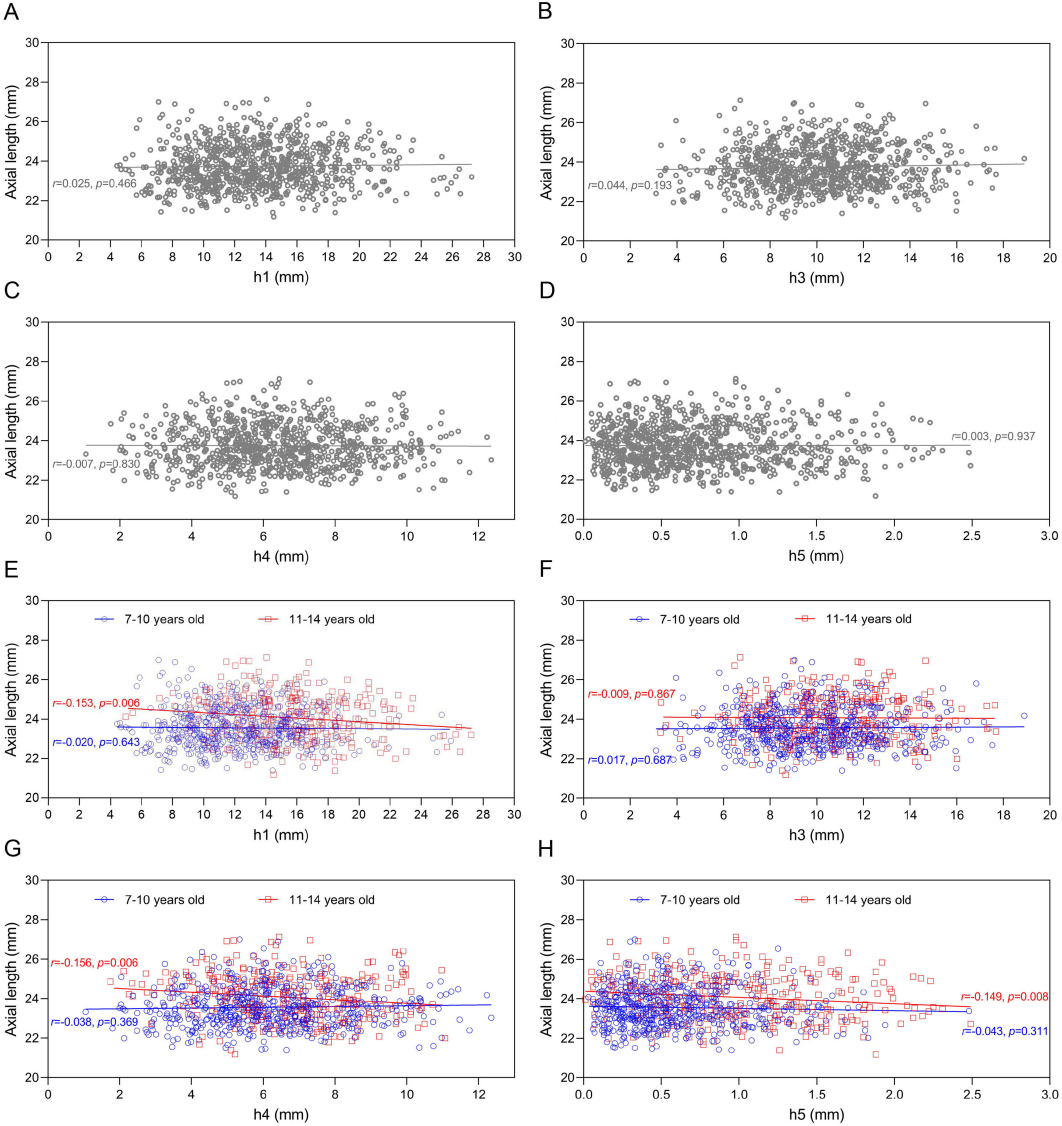
Association between axial length (AL) and pulse wave amplitude. **(A–D)** Association between AL and h1, h3, h4, and h5 in all children. **(E–H)** Association bbbbbetween AL and h1, h3, h4, and h5 in children aged 7–10 years and 11–14 years.

Correlation analyses between pulse amplitudes and ocular biometric parameters revealed distinct age-related patterns (Figures 4, 5). Among all children, pulse amplitudes correlated neither with spherical SE nor AL. However, age-stratified analyses showed that in children aged 11–14 years, higher h1 significantly correlated with greater SE (less myopic refraction; *r* = 0.193, *P* = 0.001) and shorter AL (*r* = −0.153, *P* = 0.006) (Figures 4E, 5E). A positive correlation between h1 and SE was also seen in the 7–10 years group (*r* = 0.096, *P* = 0.023) (Figure 4E). Additionally, in the older children, both h4 and h5 amplitudes were negatively correlated with AL (Figures 5G,H).

### 3.5 Associations with refractive parameters and TCM constitution scores

Generalized linear models (GLM) adjusting for the same covariates are detailed in Supplementary Tables 3–6. In the total population, no TCM parameters were significantly associated with SE or AL in the multivariate models. However, age-stratified GLM reinforced the correlation findings. Higher h1 value was independently associated with a greater SE (less myopia) in the 11–14 years group (Model 1: β = 0.131, 95% CI: 0.062–0.201, *P* < 0.001; Model 2: β = 0.132, 95% CI: 0.062–0.202, *P* < 0.001) (Supplementary Table 4). Conversely, longer AL was independently associated with lower h1 (Model 1: β = −0.083, 95% CI: −0.130 to −0.037, *P* < 0.001; Model 2: β = −0.085, 95% CI: −0.132 to −0.038, *P* < 0.001) and lower h5 (Model 1:β = −0.413, 95% CI: −0.629 to −0.197, *P* < 0.001; Model 2: β = −0.425, 95% CI: −0.642 to −0.208, *P* < 0.001) in the 11–14 years group (Supplementary Table 6). Spearman correlation analysis revealed that in myopic children, lower h1 and h4 values were significantly correlated with higher scores of *Qi stagnation, Yang deficiency, and Qi deficiency constitutions*, whereas no such correlations were observed in non-myopic children (Supplementary Table 7).

## 4 Discussion

This study pioneers the application of digital and intelligent TCM diagnostics to empirically investigate the systemic correlates of childhood myopia. Our key finding—that a lower amplitude of the main pulse wave (h1) is independently and negatively associated with myopia after adjusting for established risk factors—provides the first quantitative evidence linking objective pulse parameters to myopia in children. This observation aligns with and substantiates the TCM holistic concept of “Qi and Blood deficiency” in myopia pathogenesis (47). Importantly, while pulse diagnosis emerged as a sensitive systemic indicator, objectively quantified tongue and facial parameters did not show significant associations in our adjusted models. These results directly address the research gap identified in the introduction, demonstrating that digital TCM pulse analysis can capture systemic physiological alterations associated with myopia, whereas tongue and facial manifestations may be subtler or more context-dependent in this pediatric population.

The holistic interpretation of myopia in TCM, often framed as “deficiency of Qi and Blood failing to nourish the eyes,” has historically relied on subjective assessment. Modern pulse waveform analysis allows this theory to be examined through a hemodynamic perspective. In TCM, the pulse reflects the state of Qi and Blood (48); in biomedical terms, h1 primarily corresponds to left ventricular ejection strength and arterial compliance (49). Our finding of reduced h1 in myopic children suggests a state of diminished circulatory vigor, which may parallel the TCM notion of “Qi deficiency failing to propel Blood.” This is consistent with recent Western studies indicating associations between myopia and systemic vascular factors, such as pre-hypertension (17). Furthermore, previous studies in adults have linked lower h1 values to Yin deficiency or special constitutions compared to a balanced constitution (50, 51), and children with recurrent respiratory tract infection and lung Qi deficiency also exhibit lower pulse wave amplitudes (52). To provide more specific empirical evidence linking h1 to Qi deficiency in our pediatric cohort, we performed correlation analyses between pulse wave amplitudes and the scores of five major TCM body constitutions (including Qi deficiency). As shown in Supplementary Table 7, h1 was significantly negatively correlated with the Qi deficiency score in the total cohort (*r* = −0.419, *P* = 0.010), and this negative correlation was even stronger in the myopic subgroup (*r* = −0.476, *P* = 0.034). No such significant correlation was observed in non-myopic children. These results directly demonstrate that a lower h1 amplitude is associated with a higher degree of Qi deficiency tendency, particularly among myopic children. Thus, the lower h1 value may represent a quantifiable, systemic hemodynamic profile that accompanies myopic changes, bridging classical TCM theory with contemporary vascular physiology. Contemporary research also suggests a link between ocular blood flow, particularly choroidal perfusion, and myopia development (15, 53), hinting at a potential common pathway involving microcirculatory adequacy.

The selection of the left Guan position for pulse acquisition deserves specific discussion. In TCM, this position is linked to the Liver, which is considered closely related to visual function through its role in storing Blood (19). Our results may therefore reflect an objective correlate of “Liver Blood deficiency,” a traditional pattern associated with visual decline. From a practical perspective, the *Guan* position is the easiest site to obtain a stable pulse wave with optimal amplitude (42). Due to ongoing osteological development in children aged 7–14 years, the radial artery at the *Cun* and *Chi* positions is relatively shallow and narrow, and the distance among the three positions is narrower compared with adults (54). Moreover, most current pulse diagnosis instruments are primarily designed for adult wrists. In children, simultaneous multi-point pressure sensor positioning could therefore lead to sensor overlap or pressure interference, compromising signal consistency. Thus, single-position measurement at the left *Guan* was a pragmatic choice to ensure data quality and reproducibility. While multi-point pulse analysis might offer additional insights in future technological iterations (23, 55, 56), our standardized approach provides a reproducible and theoretically grounded foundation for this novel investigation.

Subgroup analyses revealed that the association between pulse parameters and myopia varied with age. Notably, h1 showed stronger correlations with SE and AL in older children (11–14 years), and h5 (dicrotic wave amplitude) was also negatively associated with AL in older age group. These age-related differences may reflect developmental changes in cardiovascular dynamics and vascular compliance (57). From a TCM perspective, the developmental trajectory of pulse amplitude with age aligns with the concept of “gradual abundance of Qi and Blood” during childhood, whereas the blunted increase in h1 among myopic children may reflect a state of “Qi deficiency” that becomes more pronounced with increasing age and academic strain. This is consistent with recent causal inference findings that pulse rate is negatively associated with myopia risk in children, suggesting that cardiovascular parameters are dynamically linked to refractive development and that such associations may strengthen with age (58). The observed increase in h1, h3, h4, and h5 with age further underscores that pulse characteristics evolve during childhood (59, 60), reinforcing the importance of age stratification in both study design and clinical interpretation. To explore age-specific screening thresholds, we performed ROC curve analysis and derived h1 cutoffs for each age group (Table 5). Because h1 increases significantly with age, a single fixed cutoff is inappropriate; these age-specific cutoffs may serve as reference values for current myopia status in school-based screening, but they require validation in independent longitudinal cohorts before clinical implementation.

In contrast to the significant pulse findings, objectively quantified tongue and facial parameters were not associated with myopia in adjusted models. This absence of association does not invalidate TCM tongue or face diagnosis but suggests several possible explanations. From a pathophysiological perspective, pulse waveforms reflect dynamic changes in Qi and Blood circulation and may be more sensitive to systemic alterations accompanying myopia development, whereas tongue and facial features tend to reflect more chronic, cumulative visceral imbalances. Childhood myopia typically progresses over a relatively short time course, and during this developmental period, systemic imbalances may not yet have manifested as distinct or characteristic tongue and facial changes. From a technical perspective, the DS01-B system currently quantifies tongue and facial color using the CIELAB color space. However, studies on tongue image analysis have demonstrated that different color spaces (e.g., Lab, RGB, YCrCb, HIS) capture different aspects of pathological changes (61, 62). Our system’s reliance on a single color space (CIELAB) may have limited its ability to detect subtle myopia-related changes in tongue and facial appearance. Moreover, the DS01-B system was primarily developed and validated in adult populations; its application in children—whose skin and oral mucosa have different optical properties and less accumulated pathological changes—has not been extensively studied. Thus, while pulse analysis appears to be a more sensitive systemic biomarker in this context, the role of tongue and face diagnosis in childhood myopia warrants further refinement and longitudinal observation. Future digital TCM research could improve sensitivity by integrating multiple color spaces and by developing pediatric-specific image acquisition protocols and normative databases.

Our analysis of TCM body constitutions offers a complementary perspective. Myopic children showed a lower prevalence of balanced constitution and higher rates of biased constitutions such as Qi stagnation, Yin deficiency, Yang deficiency and Qi deficiency. Although constitutional assessment in children involves certain methodological considerations, this pattern supports the view that constitutional predisposition may influence myopia susceptibility (63). Further correlation analyses revealed that reduced h1 is a shared feature across multiple biased constitutions in myopic children, particularly Qi stagnation, Yang deficiency, and Qi deficiency, which strengthens the narrative that lower pulse wave amplitude reflects a systemic state of Qi-Blood insufficiency. Moreover, after adjusting for TCM body constitution in multivariable logistic regression models, h1 remained independently and negatively associated with myopia, indicating that the association between h1 and myopia is robust and not merely driven by constitutional differences. The convergence between biased constitutions and lower h1 values suggests a possible shared pathway—insufficient nourishment of the eyes due to Qi and Blood deficiency—with pulse amplitude serving as a potential quantitative marker of this systemic deficit.

Several limitations must be acknowledged. First, the cross-sectional design precludes causal inference between pulse parameters and myopia, while the single-center nature may limit the representativeness of the sample and consequently constrain the generalizability of these observations. Therefore, longitudinal and multi-center studies are needed to validate and extend our findings. Second, while we adjusted for key confounders, self-reported behavioral data may introduce recall bias. Future studies could employ objective measures such as wearable devices to track near work and outdoor time, thereby minimizing this bias. Third, the single-position (*Guan*) pulse measurement, though justified, may not capture all visceral information relevant to myopia. Nevertheless, future studies employing multi-position pulse acquisition, enabled by advances in sensor miniaturization and pediatric-specific devices, would provide more comprehensive systemic information. Fourth, although the digital TCM instruments (DS01-DI and DS01-B) have demonstrated good reliability and validity in adult populations, further validation specifically in pediatric populations is warranted, as the applicability of these devices to children has not been extensively studied. Finally, the age range (7–14 years) may limit the generalizability of findings to younger or older populations.

## 5 Conclusion

This study demonstrates that digital TCM pulse diagnosis can detect systemic physiological alterations associated with childhood myopia, empirically supporting the holistic view that myopia involves more than local ocular changes. The significant association with pulse wave amplitude—but not with tongue or facial features—highlights the specificity and sensitivity of vascular dynamics in reflecting myopia-related systemic status. These findings advance a convergent framework that integrates TCM systemic diagnostics with modern ophthalmology, suggesting that combined ocular and systemic monitoring could enhance early risk identification and holistic management strategies for myopia prevention. Future research should explore longitudinal and mechanistic links between fundus blood flow and digital TCM parameters.

## Data Availability

The original contributions presented in this study are included in the article/Supplementary material, further inquiries can be directed to the corresponding authors.

## Footnotes

1
www.openepi.com
