# Supplementary material for: Objective pulse wave amplitude as a potential systemic correlate in childhood myopia: a digital TCM-based study
Source: Front Med (Lausanne). 2026 May 8;13:1815599. doi: 10.3389/fmed.2026.1815599 (PMC13194474; doi:10.3389/fmed.2026.1815599)
Supplement: Supplementary file 1 [file Supplementary_file_1.docx]

Supplementary Material

# Supplementary Table

Supplementary Table 1. The meaning of basic parameters in pulse picture.

| Parameters | Meaning |
| --- | --- |
| h1 | Main wave amplitude. It reflects the compliance of the aorta and the cardiac ejection function of the left ventricular. |
| h3 | Front wave amplitude of dicrotic pulse. It reflects the elasticity of arterial vessels and its peripheral resistance. |
| h4 | Dicrotic notch amplitude. It reflects the peripheral resistance of arterial vessels and the closure of aortic valve. |
| h5 | Dicrotic pulse amplitude. It reflects the compliance of the aorta and the function of aortic valve. |
| t4 | Left ventricular systolic duration. The time value from the start point to the dicrotic notch on the pulse picture. |
| t5 | Left ventricular diastolic duration. The time value from the dicrotic notch to the end point on the pulse picture. |
| t | Left ventricular systolic and diastolic duration. The time value from the start point to the end point on the pulse picture. |
| h3/h1 | Front wave amplitude of dicrotic pulse- to- main wave amplitude ratio. It reflects the compliance of the aorta and its peripheral resistance. |
| h4/h1 | Dicrotic notch amplitude - to- main wave amplitude ratio. It reflects the peripheral resistance of arterial vessels. |
| h5/h1 | Dicrotic pulse amplitude - to- main wave amplitude ratio. It reflects the compliance of the aorta and the function of aortic valve. |
| As | Systolic area. The area on the pulse picture is related to cardiac output. |
| Ad | Diastolic area. |

Supplementary Table 2. Age-stratified univariate logistic regression for pulse, tongue, and face diagnoses and myopia.

| Variables | 7-10 years old (n=557) | | |  | 11-14 years old (n=316) | | |
| --- | --- | --- | --- | --- | --- | --- | --- |
|  | OR (95% CI) | *P* value | *P*-FDR |  | OR (95% CI) | *P* value | *P*-FDR |
| Age (years) | 1.828 (1.537, 2.175) | ＜0.001 | ＜0.001 |  | 1.484 (1.224, 1.800) | ＜0.001 | 0.005 |
| Sex, female | 0.993 (0.712, 1.386) | 0.969 | 0.993 |  | 0.903 (0.567, 1.437) | 0.666 | 0.799 |
| BMI (kg/m^2^) | 1.001 (0.953, 1.051) | 0.974 | 0.986 |  | 1.045 (0.986, 1.109) | 0.138 | 0.297 |
| Number of myopic parents |  |  |  |  |  |  |  |
| 0 | Ref. |  |  |  | Ref. |  |  |
| 1 | 1.664 (1.106, 2.504) | 0.015 | 0.252 |  | 1.494 (0.870, 2.563) | 0.146 | 0.307 |
| 2 | 1.577 (1.020, 2.440) | 0.041 | 0.431 |  | 1.948 (1.071, 3.545) | 0.029 | 0.097 |
| Indoor near work time per day  on weekdays (hours) |  |  |  |  |  |  |  |
| ＜4 | Ref. |  |  |  | Ref. |  |  |
| ≥4 to＜8 | 1.171 (0.818, 1.677) | 0.388 | 0.815 |  | 1.267 (0.693, 2.317) | 0.443 | 0.631 |
| ≥8 | 1.994 (1.151, 3.454) | 0.014 | 0.294 |  | 1.436 (0.717, 2.877) | 0.308 | 0.479 |
| Indoor near work time per day  on weekends (hours) |  |  |  |  |  |  |  |
| ＜4 | Ref. |  |  |  | Ref. |  |  |
| ≥4 to＜8 | 1.044 (0.736, 1.481) | 0.811 | 1.000 |  | 1.200 (0.688, 2.093) | 0.521 | 0.673 |
| ≥8 | 1.313 (0.711, 2.426) | 0.385 | 0.829 |  | 1.104 (0.568, 2.146) | 0.771 | 0.864 |
| Outdoor activity time per day  on weekdays (hours) |  |  |  |  |  |  |  |
| ＜1 | Ref. |  |  |  | Ref. |  |  |
| ≥1 to＜2 | 0.704 (0.485, 1.020) | 0.064 | 0.538 |  | 0.610 (0.365, 1.020) | 0.065 | 0.182 |
| ≥2  Outdoor activity time per day  on weekends (hours) | 0.747 (0.471, 1.186) | 0.217 | 0.959 |  | 0.351 (0.173, 0.712) | 0.004 | 0.022 |
| ＜1 | Ref. |  |  |  | Ref. |  |  |
| ≥1 to＜2 | 0.657 (0.428, 1.011) | 0.056 | 0.523 |  | 0.647 (0.374, 1.121) | 0.120 | 0.272 |
| ≥2 | 0.780 (0511, 1.192) | 0.251 | 0.878 |  | 0.315 (0.170, 0.586) | ＜0.001 | 0.006 |
| Body constitution type, n (%) |  |  |  |  |  |  |  |
| Balance | Ref. |  |  |  | Ref. |  |  |
| Qi stagnation | 2.318 (1.444, 3.721) | 0.001 | 0.030 |  | 1.552 (0.869, 2.773) | 0.138 | 0.315 |
| Yin deficiency | 2.377 (1.477, 3.827) | ＜0.001 | 0.016 |  | 1.908 (0.715, 5.093) | 0.197 | 0.390 |
| Yang deficiency | 2.041 (1.061, 3.924) | 0.033 | 0.367 |  | 1.886 (0.648, 5.485) | 0.244 | 0.426 |
| Qi deficiency | 1.565 (0.709, 3.458) | 0.268 | 0.745 |  | 1.571 (0.392, 6.297) | 0.523 | 0.665 |
| Other types^a^ | 1.180 (0.436, 3.193) | 0.745 | 0.990 |  | 1.636 (0.643, 4.162) | 0.302 | 0.489 |
| **Pulse condition** |  |  |  |  |  |  |  |
| h1 (mm) | 0.927 (0.883, 0.974) | 0.002 | 0.045 |  | 0.883 (0.828, 0.941) | ＜0.001 | 0.003 |
| h3 (mm) | 0.953 (0.897, 1.013) | 0.120 | 0.840 |  | 0.879 (0.803, 0.961) | 0.005 | 0.025 |
| h4 (mm) | 0.963 (0.886, 1.046) | 0.370 | 0.841 |  | 0.816 (0.718, 0.928) | 0.002 | 0.014 |
| h5 (mm) | 0.639 (0.419, 0.974) | 0.037 | 0.518 |  | 0.655 (0.431, 0.996) | 0.048 | 0.144 |
| t4 (s) | 1.661 (0.100, 27.504) | 0.723 | 0.996 |  | 1.684 (0.023, 123.033) | 0.812 | 0.886 |
| t5 (s) | 1.307 (0.316, 5.403) | 0.711 | 0.995 |  | 1.067 (0.085, 13.348) | 0.960 | 0.972 |
| t (s) | 1.346 (0.394, 4.590) | 0.635 | 1.000 |  | 0.733 (0.108, 4.984) | 0.751 | 0.853 |
| h3/h1 | 2.589 (1.058, 6.334) | 0.037 | 0.518 |  | 2.335 (0.565, 9.651) | 0.241 | 0.422 |
| h4/h1 | 4.698 (1.383, 15.963) | 0.013 | 0.364 |  | 1.127 (0.248, 5.111) | 0.877 | 0.945 |
| h5/h1 | 0.229 (0.004, 13.352) | 0.477 | 0.871 |  | 0.003 (0.001, 5.883) | 0.134 | 0.296 |
| As | 0.997 (0.989, 1.005) | 0.410 | 0.801 |  | 0.977 (0.964, 0.990) | ＜0.001 | 0.005 |
| Ad | 0.996 (0.984, 1.008) | 0.519 | 0.908 |  | 0.983 (0.969, 0.998) | 0.024 | 0.092 |
| **Tongue color** |  |  |  |  |  |  |  |
| All-L | 0.999 (0.945, 1.055) | 0.965 | 1.000 |  | 0.918 (0.849, 0.992) | 0.031 | 0.100 |
| All-a | 1.012 (0.929, 1.102) | 0.788 | 1.000 |  | 0.936 (0.832, 1.053) | 0.271 | 0.465 |
| All-b | 0.974 (0.874, 1.086) | 0.638 | 1.000 |  | 0.849 (0.740, 0.974) | 0.020 | 0.080 |
| Middle-L | 0.998 (0.952, 1.046) | 0.938 | 1.000 |  | 0.946 (0.884, 1.013) | 0.110 | 0.257 |
| Middle-a | 1.036 (0.973, 1.103) | 0.271 | 0.785 |  | 0.966 (0.884, 1.055) | 0.440 | 0.648 |
| Middle-b | 0.987 (0.899, 1.084) | 0.788 | 1.000 |  | 0.895 (0.794, 1.009) | 0.070 | 0.190 |
| Root-L | 1.006 (0.973, 1.040) | 0.707 | 1.000 |  | 0.977 (0.934, 1.022) | 0.304 | 0.482 |
| Root-a | 0.999 (0.933, 1.071) | 0.985 | 0.985 |  | 0.939 (0.857, 1.029) | 0.179 | 0.358 |
| Root-b | 0.945 (0.864, 1.035) | 0.225 | 0.900 |  | 0.925 (0.826, 1.035) | 0.175 | 0.359 |
| Right-L | 0.991 (0.939, 1.047) | 0.754 | 0.989 |  | 0.934 (0.867, 1.007) | 0.075 | 0.197 |
| Right-a | 0.993 (0.912, 1.082) | 0.875 | 1.000 |  | 0.980 (0.872, 1.102) | 0.737 | 0.848 |
| Right-b | 0.982 (0.882, 1.094) | 0.742 | 1.000 |  | 0.871 (0.761, 0.996) | 0.044 | 0.137 |
| Left-L | 1.003 (0.953, 1.056) | 0.916 | 1.000 |  | 0.931 (0.866, 1.001) | 0.053 | 0.154 |
| Left-a | 1.008 (0.927, 1.095) | 0.856 | 1.000 |  | 0.962 (0.855, 1.082) | 0.516 | 0.699 |
| Left-b | 0.957 (0.861, 1.064) | 0.417 | 0.796 |  | 0.858 (0.751, 0.981) | 0.025 | 0.091 |
| Tip-L | 0.999 (0.945, 1.055) | 0.966 | 1.000 |  | 0.938 (0.870, 1.011) | 0.093 | 0.230 |
| Tip-a | 1.013 (0.954, 1.075) | 0.677 | 0.997 |  | 0.928 (0.851, 1.011) | 0.089 | 0.227 |
| Tip-b | 1.048 (0.950, 1.156) | 0.353 | 0.872 |  | 0.808 (0.707, 0.923) | 0.002 | 0.014 |
| **Tongue shape** |  |  |  |  |  |  |  |
| Medium | Ref. |  |  |  | Ref. |  |  |
| Swollen | 1.240 (0.848, 1.815) | 0.267 | 0.801 |  | 0.964 (0.575, 1.615) | 0.889 | 0.934 |
| Thin | 0.864 (0.515, 1.448) | 0.579 | 0.993 |  | 1.172 (0.540, 2.543) | 0.688 | 0.814 |
| **Tongue coating color** |  |  |  |  |  |  |  |
| All-L | 0.998 (0.987, 1.010) | 0.783 | 1.000 |  | 0.993 (0.975, 1.011) | 0.444 | 0.622 |
| All-a | 0.992 (0.953, 1.031) | 0.669 | 1.000 |  | 0.977 (0.921, 1.037) | 0.441 | 0.639 |
| All-b | 0.989 (0.937, 1.043) | 0.686 | 0.994 |  | 0.965 (0.888, 1.049) | 0.408 | 0.612 |
| Middle-L | 0.996 (0.988, 1.004) | 0.373 | 0.824 |  | 1.002 (0.988, 1.015) | 0.804 | 0.889 |
| Middle-a | 0.984 (0.955, 1.015) | 0.313 | 0.848 |  | 1.015 (0.971, 1.061) | 0.512 | 0.705 |
| Middle-b | 0.982 (0.942, 1.024) | 0.405 | 0.810 |  | 1.005 (0.937, 1.079) | 0.886 | 0.942 |
| Root-L | 0.995 (0.986, 1.005) | 0.320 | 0.840 |  | 0.996 (0.984, 1.009) | 0.564 | 0.707 |
| Root-a | 0.979 (0.947, 1.013) | 0.217 | 0.959 |  | 0.980 (0.935, 1.027) | 0.392 | 0.599 |
| Root-b | 0.977 (0.938, 1.018) | 0.262 | 0.815 |  | 0.984 (0.930, 1.041) | 0.577 | 0.713 |
| Right-L | 1.001 (0.990, 1.011) | 0.899 | 1.000 |  | 0.990 (0.974, 1.006) | 0.229 | 0.418 |
| Right-a | 1.002 (0.968, 1.037) | 0.920 | 1.000 |  | 0.965 (0.916, 1.017) | 0.186 | 0.363 |
| Right-b | 1.001 (0.954, 1.051) | 0.962 | 1.000 |  | 0.952 (0.883, 1.027) | 0.207 | 0.395 |
| Left-L | 0.997 (0.987, 1.008) | 0.590 | 0.991 |  | 0.991 (0.975, 1.007) | 0.286 | 0.481 |
| Left-a | 0.985 (0.952, 1.020) | 0.401 | 0.822 |  | 0.973 (0.924, 1.024) | 0.297 | 0.489 |
| Left-b | 0.983 (0.936, 1.031) | 0.476 | 0.888 |  | 0.961 (0.892, 1.036) | 0.303 | 0.490 |
| Tip-L | 1.010 (0.997, 1.022) | 0.120 | 0.840 |  | 1.005 (0.987, 1.023) | 0.596 | 0.726 |
| Tip-a | 1.029 (0.990, 1.070) | 0.143 | 0.858 |  | 1.017 (0.962, 1.075) | 0.549 | 0.699 |
| Tip-b | 1.044 (0.982, 1.111) | 0.167 | 0.825 |  | 1.006 (0.918, 1.102) | 0.896 | 0.929 |
| **Facial color** |  |  |  |  |  |  |  |
| All-L | 0.975 (0.905, 1.052) | 0.516 | 0.922 |  | 0.801 (0.708, 0.907) | ＜0.001 | 0.005 |
| All-a | 0.980 (0.867, 1.108) | 0.747 | 0.996 |  | 0.943 (0.789, 1.126) | 0.516 | 0.699 |
| All-b | 0.934 (0.833, 1.047) | 0.242 | 0.884 |  | 0.767 (0.660, 0.892) | 0.001 | 0.008 |
| Forehead-L | 0.979 (0.935, 1.025) | 0.369 | 0.861 |  | 0.913 (0.850, 0.982) | 0.014 | 0.062 |
| Forehead-a | 0.949 (0.859, 1.048) | 0.298 | 0.834 |  | 0.917 (0.799, 1.053) | 0.221 | 0.413 |
| Forehead-b | 0.944 (0.855, 1.042) | 0.254 | 0.853 |  | 0.920 (0.802, 1.057) | 0.239 | 0.427 |
| Right cheek-L | 0.984 (0.917, 1.055) | 0.647 | 1.000 |  | 0.873 (0.782, 0.976) | 0.017 | 0.071 |
| Right cheek-a | 0.993 (0.906, 1.089) | 0.884 | 1.000 |  | 0.992 (0.867, 1.136) | 0.909 | 0.931 |
| Right cheek-b | 0.953 (0.877, 1.036) | 0.257 | 0.830 |  | 0.837 (0.758, 0.925) | ＜0.001 | 0.005 |
| Left cheek-L | 1.016 (0.953, 1.084) | 0.630 | 1.000 |  | 0.902 (0.824, 0.987) | 0.025 | 0.091 |
| Left cheek-a | 1.020 (0.930, 1.118) | 0.673 | 1.000 |  | 1.027 (0.899, 1.173) | 0.691 | 0.806 |
| Left cheek-b | 0.947 (0.870, 1.032) | 0.216 | 1.000 |  | 0.831 (0.750, 0.921) | ＜0.001 | 0.006 |
| Periocular-L | 1.004 (0.943, 1.069) | 0.893 | 1.000 |  | 0.891 (0.816, 0.972) | 0.010 | 0.047 |
| Periocular-a | 0.922 (0.805, 1.056) | 0.241 | 0.920 |  | 0.937 (0.770, 1.141) | 0.518 | 0.680 |
| Periocular-b | 0.920 (0.830, 1.021) | 0.115 | 0.878 |  | 0.773 (0.671, 0.889) | ＜0.001 | 0.006 |
| Nose-L | 1.009 (0.935, 1.089) | 0.815 | 0.992 |  | 0.776 (0.685, 0.879) | ＜0.001 | 0.003 |
| Nose-a | 0.917 (0.816, 1.031) | 0.149 | 0.834 |  | 1.001 (0.853, 1.173) | 0.993 | 0.993 |
| Nose-b | 0.959 (0.878, 1.049) | 0.362 | 0.869 |  | 0.829 (0.741, 0.929) | 0.001 | 0.008 |
| Lip-L | 0.958 (0.905, 1.015) | 0.149 | 0.834 |  | 0.863 (0.781, 0.954) | 0.004 | 0.022 |
| Lip-a | 1.005 (0.914, 1.104) | 0.922 | 1.000 |  | 0.881 (0.755, 1.029) | 0.109 | 0.262 |
| Lip-b | 0.949 (0.853, 1.057) | 0.341 | 0.868 |  | 0.782 (0.670, 0.913) | 0.002 | 0.014 |

^a^ Other types include Special constitution, Damp-heat constitution, Phlegm dampness constitution, and Blood-stasis constitution.

The color variables (L, a, b) were defined in terms of the CIELAB space, in which L (lightness), a (red-green axis), b (Yellow-blue axis). BMI, body mass index;

OR, Odds ratio; CI, Confidence interval; FDR, false discovery rate.

Supplementary Table 3. Age-stratified univariate analysis of pulse, tongue, and face diagnoses in association with spherical equivalent.

| Variables | Total (n=873) | | | |  | 7-10 years old (n=557) | | | |  | 11-14 years old (n=316) | | | |
| --- | --- | --- | --- | --- | --- | --- | --- | --- | --- | --- | --- | --- | --- | --- |
|  | *β* | 95% CI | *P* value | *P*-FDR |  | *β* | 95% CI | *P* value | *P*-FDR |  | *β* | 95% CI | *P* value | *P*-FDR |
| Age (years) | -0.328 | -0.386 to -0.271 | ＜0.001 | ＜0.001 |  | -0.505 | -0.647 to -0.364 | ＜0.001 | ＜0.001 |  | -0.482 | -0.665 to -0.299 | ＜0.001 | ＜0.001 |
| BMI (kg/m^2^) | -0.066 | -0.101 to -0.030 | ＜0.001 | 0.002 |  | -0.006 | -0.052 to 0.040 | 0.800 | 0.913 |  | -0.064 | -0.122 to -0.006 | 0.031 | 0.079 |
| Sex |  |  |  |  |  |  |  |  |  |  |  |  |  |  |
| male | Ref. |  |  |  |  | Ref. |  |  |  |  | Ref. |  |  |  |
| female | 0.015 | -0.255 to 0.285 | 0.914 | 0.935 |  | 0.119 | -0.191 to 0.429 | 0.451 | 0.854 |  | -0.070 | -0.546 to 0.405 | 0.772 | 0.848 |
| Number of myopic parents |  |  |  |  |  |  |  |  |  |  |  |  |  |  |
| 0 | Ref. |  |  |  |  | Ref. |  |  |  |  | Ref. |  |  |  |
| 1 | -0.454 | -0.772 to -0.136 | 0.005 | 0.014 |  | -0.483 | -0.855 to -0.111 | 0.011 | 0.326 |  | -0.745 | -1.289 to -0.202 | 0.007 | 0.027 |
| 2 | -0.533 | -0.873 to -0.192 | 0.002 | 0.007 |  | -0.421 | -0.820 to -0.023 | 0.038 | 0.307 |  | -1.054 | -1.633 to -0.475 | ＜0.001 | 0.003 |
| Indoor near work time per day  on weekdays (hours) |  |  |  |  |  |  |  |  |  |  |  |  |  |  |
| ＜4 | Ref. |  |  |  |  | Ref. |  |  |  |  | Ref. |  |  |  |
| ≥4 to＜8 | -0.383 | -0.686 to -0.080 | 0.013 | 0.026 |  | 0.008 | -0.324 to 0.339 | 0.963 | 0.974 |  | -0.629 | -1.252 to -0.005 | 0.048 | 0.102 |
| ≥8 | -0.429 | -0.830 to -0.028 | 0.036 | 0.065 |  | -0.435 | -0.937 to 0.068 | 0.090 | 0.471 |  | 0.039 | -0.669 to 0.746 | 0.915 | 0.958 |
| Indoor near work time per day  on weekends (hours) |  |  |  |  |  |  |  |  |  |  |  |  |  |  |
| ＜4 | Ref. |  |  |  |  | Ref. |  |  |  |  | Ref. |  |  |  |
| ≥4 to＜8 | -0.422 | -0.710 to -0.133 | 0.004 | 0.012 |  | -0.122 | -0.446 to 0.203 | 0.463 | 0.858 |  | -0.511 | -1.083 to 0.060 | 0.080 | 0.151 |
| ≥8 | -0.702 | -1.121 to -0.283 | 0.001 | 0.005 |  | -0.296 | -0.872 to 0.281 | 0.315 | 0.701 |  | -0.500 | -1.181 to 0.181 | 0.150 | 0.234 |
| Outdoor activity time per day  on weekdays (hours) |  |  |  |  |  |  |  |  |  |  |  |  |  |  |
| ＜1 | Ref. |  |  |  |  | Ref. |  |  |  |  | Ref. |  |  |  |
| ≥1 to＜2 | 0.538 | 0.244 to 0.832 | ＜0.001 | 0.002 |  | 0.428 | 0.085 to 0.770 | 0.014 | 0.312 |  | 0.490 | -0.022 to 1.001 | 0.061 | 0.123 |
| ≥2  Outdoor activity time per day  on weekends (hours) | 0.769 | 0.388 to 1.151 | ＜0.001 | 0.001 |  | 0.421 | -0.005 to 0.847 | 0.053 | 0.363 |  | 1.125 | 0.395 to 1.855 | 0.003 | 0.013 |
| ＜1 | Ref. |  |  |  |  | Ref. |  |  |  |  | Ref. |  |  |  |
| ≥1 to＜2 | 0.448 | 0.122 to 0.774 | 0.007 | 0.016 |  | 0.447 | 0.050 to 0.844 | 0.027 | 0.300 |  | 0.218 | -0.314 to 0.750 | 0.422 | 0.569 |
| ≥2 | 0.728 | 0.391 to 1.065 | ＜0.001 | 0.001 |  | 0.385 | -0.007 to 0.777 | 0.054 | 0.343 |  | 0.822 | 0.257 to 1.508 | 0.006 | 0.024 |
| Body constitution type, n (%) |  |  |  |  |  |  |  |  |  |  |  |  |  |  |
| Balance | Ref. |  |  |  |  | Ref. |  |  |  |  | Ref. |  |  |  |
| Qi stagnation | -0.707 | -1.062 to -0.352 | ＜0.001 | 0.001 |  | -0.708 | -1.136 to -0.280 | 0.001 | 0.045 |  | -0.517 | -1.097 to 0.064 | 0.081 | 0.147 |
| Yin deficiency | -0.232 | -0.642 to 0.179 | 0.269 | 0.374 |  | -0.481 | -0.911 to -0.051 | 0.028 | 0.277 |  | -0.164 | -1.096 to 0.767 | 0.730 | 0.822 |
| Yang deficiency | -0.422 | -0.961 to 0.118 | 0.126 | 0.197 |  | -0.359 | -0.956 to 0.238 | 0.238 | 0.683 |  | -0.757 | -1.771 to 0.257 | 0.143 | 0.227 |
| Qi deficiency | -0.615 | -1.291 to 0.061 | 0.075 | 0.128 |  | -0.776 | -1.503 to -0.049 | 0.037 | 0.329 |  | -0.574 | -1.937 to 0.788 | 0.409 | 0.560 |
| Other types^a^ | -0.537 | -1.182 to 0.107 | 0.102 | 0.171 |  | -0.222 | -1.123 to 0.680 | 0.630 | 0.890 |  | -0.354 | -1.268 to 0.560 | 0.448 | 0.595 |
| **Pulse condition** |  |  |  |  |  |  |  |  |  |  |  |  |  |  |
| h1 | 0.011 | -0.023 to 0.045 | 0.528 | 0.618 |  | 0.052 | 0.007 to 0.097 | 0.022 | 0.392 |  | 0.107 | 0.047 to 0.168 | ＜0.001 | 0.004 |
| h3 | 0.009 | -0.041 to 0.060 | 0.720 | 0.781 |  | 0.020 | -0.038 to 0.078 | 0.500 | 0.873 |  | 0.082 | -0.010 to 0.173 | 0.080 | 0.151 |
| h4 | 0.024 | -0.045 to 0.093 | 0.491 | 0.583 |  | 0.003 | -0.074 to 0.081 | 0.936 | 0.969 |  | 0.157 | 0.031 to 0.283 | 0.015 | 0.045 |
| h5 | -0.151 | -0.431 to 0.128 | 0.289 | 0.396 |  | 0.219 | -0.184 to 0.623 | 0.287 | 0.730 |  | 0.345 | -0.092 to 0.782 | 0.122 | 0.197 |
| t4 | 2.399 | 0.064 to 4.733 | 0.044 | 0.078 |  | 3.062 | 0.440 to 5.684 | 0.022 | 0.392 |  | 0.091 | -4.170 to 4.351 | 0.967 | 0.989 |
| t5 | -0.798 | -2.063 to 0.468 | 0.217 | 0.317 |  | -0.263 | -1.630 to 1.103 | 0.706 | 0.885 |  | -0.402 | -2.987 to 2.183 | 0.761 | 0.847 |
| t | -0.470 | -1.505 to 0.565 | 0.373 | 0.481 |  | -0.193 | -1.336 to 0.950 | 0.741 | 0.879 |  | 0.028 | -1.957 to 2.012 | 0.978 | 0.989 |
| h3/h1 | 0.216 | -0.483 to 0.915 | 0.545 | 0.630 |  | -0.412 | -1.213 to 0.389 | 0.313 | 0.733 |  | -0.822 | -2.198 to 0.555 | 0.242 | 0.342 |
| h4/h1 | -0.238 | -1.082 to 0.605 | 0.580 | 0.662 |  | -0.582 | -1.689 to 0.525 | 0.303 | 0.729 |  | -0.346 | -1.941 to 1.250 | 0.671 | 0.776 |
| h5/h1 | -1.831 | -5.235 to 1.572 | 0.292 | 0.394 |  | 0.691 | -3.066 to 4.447 | 0.719 | 0.877 |  | 0.265 | -5.842 to 6.373 | 0.932 | 0.965 |
| As | -0.001 | -0.008 to 0.006 | 0.951 | 0.951 |  | 0.001 | -0.007 to 0.008 | 0.847 | 0.919 |  | 0.017 | 0.005 to 0.030 | 0.005 | 0.021 |
| Ad | -0.001 | -0.010 to 0.008 | 0.821 | 0.870 |  | -0.002 | -0.014 to 0.009 | 0.704 | 0.895 |  | 0.019 | 0.004 to 0.034 | 0.014 | 0.045 |
| **Tongue color** |  |  |  |  |  |  |  |  |  |  |  |  |  |  |
| All-L | 0.058 | 0.014 to 0.102 | 0.009 | 0.019 |  | -0.003 | -0.055 to 0.048 | 0.894 | 0.947 |  | 0.135 | 0.060 to 0.210 | ＜0.001 | 0.003 |
| All-a | -0.004 | -0.072 to 0.065 | 0.921 | 0.931 |  | -0.037 | -0.116 to 0.042 | 0.358 | 0.759 |  | 0.046 | -0.074 to 0.166 | 0.452 | 0.592 |
| All-b | 0.170 | 0.089 to 0.251 | ＜0.001 | 0.000 |  | 0.006 | -0.095 to 0.107 | 0.907 | 0.950 |  | 0.235 | 0.100 to 0.369 | 0.001 | 0.007 |
| Middle-L | 0.043 | 0.005 to 0.081 | 0.025 | 0.046 |  | -0.005 | -0.049 to 0.039 | 0.819 | 0.900 |  | 0.083 | 0.016 to 0.149 | 0.014 | 0.045 |
| Middle-a | -0.022 | -0.073 to 0.028 | 0.387 | 0.492 |  | -0.034 | -0.092 to 0.024 | 0.253 | 0.704 |  | 0.010 | -0.080 to 0.100 | 0.827 | 0.898 |
| Middle-b | 0.121 | 0.051 to 0.192 | 0.001 | 0.005 |  | 0.008 | -0.078 to 0.095 | 0.848 | 0.909 |  | 0.154 | 0.034 to 0.273 | 0.012 | 0.043 |
| Root-L | 0.006 | -0.020 to 0.033 | 0.652 | 0.725 |  | -0.016 | -0.047 to 0.015 | 0.314 | 0.717 |  | 0.038 | -0.007 to 0.083 | 0.095 | 0.163 |
| Root-a | -0.008 | -0.063 to 0.047 | 0.770 | 0.826 |  | -0.036 | -0.101 to 0.028 | 0.266 | 0.717 |  | 0.029 | -0.064 to 0.123 | 0.535 | 0.661 |
| Root-b | 0.116 | 0.048 to 0.185 | 0.001 | 0.005 |  | 0.025 | -0.059 to 0.109 | 0.560 | 0.874 |  | 0.144 | 0.030 to 0.258 | 0.013 | 0.043 |
| Right-L | 0.062 | 0.019 to 0.106 | 0.005 | 0.014 |  | 0.013 | -0.038 to 0.064 | 0.618 | 0.902 |  | 0.123 | 0.050 to 0.196 | 0.001 | 0.007 |
| Right-a | -0.005 | -0.074 to 0.064 | 0.881 | 0.912 |  | -0.038 | -0.117 to 0.042 | 0.350 | 0.760 |  | 0.039 | -0.081 to 0.159 | 0.522 | 0.654 |
| Right-b | 0.169 | 0.089 to 0.248 | ＜0.001 | 0.000 |  | 0.003 | -0.097 to 0.104 | 0.949 | 0.971 |  | 0.230 | 0.097 to 0.363 | 0.001 | 0.007 |
| Left-L | 0.047 | 0.006 to 0.088 | 0.024 | 0.045 |  | -0.011 | -0.059 to 0.037 | 0.658 | 0.874 |  | 0.111 | 0.042 to 0.181 | 0.002 | 0.009 |
| Left-a | -0.006 | -0.074 to 0.061 | 0.852 | 0.892 |  | -0.031 | -0.108 to 0.046 | 0.432 | 0.854 |  | 0.025 | -0.095 to 0.145 | 0.685 | 0.782 |
| Left-b | 0.173 | 0.094 to 0.251 | ＜0.001 | 0.000 |  | 0.018 | -0.080 to 0.117 | 0.715 | 0.884 |  | 0.226 | 0.095 to 0.358 | 0.001 | 0.007 |
| Tip-L | 0.060 | 0.017 to 0.103 | 0.007 | 0.016 |  | 0.007 | -0.044 to 0.059 | 0.778 | 0.899 |  | 0.095 | 0.021 to 0.169 | 0.012 | 0.043 |
| Tip-a | 0.013 | -0.036 to 0.062 | 0.605 | 0.682 |  | -0.011 | -0.066 to 0.045 | 0.703 | 0.907 |  | 0.041 | -0.046 to 0.128 | 0.358 | 0.498 |
| Tip-b | 0.108 | 0.032 to 0.183 | 0.005 | 0.014 |  | -0.050 | -0.141 to 0.041 | 0.285 | 0.746 |  | 0.212 | 0.084 to 0.340 | 0.001 | 0.007 |
| **Tongue shape** |  |  |  |  |  |  |  |  |  |  |  |  |  |  |
| Medium | Ref. |  |  |  |  | Ref. |  |  |  |  | Ref. |  |  |  |
| Swollen | -0.360 | -0.666 to 0.055 | 0.021 | 0.041 |  | -0.329 | -0.682 to 0.024 | 0.068 | 0.378 |  | -0.380 | -0.909 to 0.149 | 0.159 | 0.240 |
| Thin | -0.097 | -0.520 to 0.326 | 0.654 | 0.719 |  | 0.060 | -0.415 to 0.534 | 0.805 | 0.907 |  | -0.523 | -1.296 to 0.250 | 0.185 | 0.274 |
| **Fur color** |  |  |  |  |  |  |  |  |  |  |  |  |  |  |
| All-L | 0.012 | 0.003 to 0.022 | 0.012 | 0.025 |  | 0.003 | -0.007 to 0.014 | 0.527 | 0.885 |  | 0.015 | -0.004 to 0.034 | 0.115 | 0.190 |
| All-a | 0.041 | 0.009 to 0.074 | 0.012 | 0.025 |  | 0.013 | -0.023 to 0.050 | 0.473 | 0.859 |  | 0.044 | -0.017 to 0.105 | 0.158 | 0.242 |
| All-b | 0.064 | 0.019 to 0.108 | 0.005 | 0.014 |  | 0.015 | -0.035 to 0.065 | 0.558 | 0.903 |  | 0.081 | -0.005 to 0.167 | 0.065 | 0.129 |
| Middle-L | 0.006 | -0.001 to 0.013 | 0.106 | 0.175 |  | 0.004 | -0.004 to 0.012 | 0.291 | 0.719 |  | 0.001 | -0.013 to 0.015 | 0.894 | 0.947 |
| Middle-a | 0.014 | -0.011 to 0.039 | 0.267 | 0.377 |  | 0.018 | -0.010 to 0.046 | 0.216 | 0.663 |  | -0.012 | -0.057 to 0.033 | 0.609 | 0.732 |
| Middle-b | 0.035 | -0.001 to 0.071 | 0.052 | 0.091 |  | 0.018 | -0.021 to 0.057 | 0.360 | 0.745 |  | 0.018 | -0.054 to 0.090 | 0.627 | 0.734 |
| Root-L | 0.011 | 0.004 to 0.019 | 0.002 | 0.007 |  | 0.007 | -0.002 to 0.016 | 0.108 | 0.506 |  | 0.011 | -0.002 to 0.023 | 0.106 | 0.178 |
| Root-a | 0.047 | 0.020 to 0.073 | 0.001 | 0.005 |  | 0.030 | -0.002 to 0.061 | 0.063 | 0.374 |  | 0.041 | -0.006 to 0.088 | 0.090 | 0.157 |
| Root-b | 0.054 | 0.022 to 0.087 | 0.001 | 0.005 |  | 0.031 | -0.007 to 0.068 | 0.111 | 0.494 |  | 0.050 | -0.008 to 0.107 | 0.089 | 0.158 |
| Right-L | 0.013 | 0.004 to 0.022 | 0.004 | 0.012 |  | 0.003 | -0.007 to 0.013 | 0.559 | 0.888 |  | 0.017 | 0.001 to 0.034 | 0.046 | 0.100 |
| Right-a | 0.042 | 0.014 to 0.070 | 0.003 | 0.010 |  | 0.008 | -0.024 to 0.040 | 0.614 | 0.911 |  | 0.056 | 0.002 to 0.110 | 0.043 | 0.096 |
| Right-b | 0.063 | 0.023 to 0.103 | 0.002 | 0.007 |  | 0.011 | -0.034 to 0.056 | 0.638 | 0.887 |  | 0.089 | 0.010 to 0.167 | 0.027 | 0.075 |
| Left-L | 0.012 | 0.004 to 0.021 | 0.006 | 0.014 |  | 0.002 | -0.007 to 0.012 | 0.642 | 0.879 |  | 0.017 | 0.001 to 0.035 | 0.049 | 0.101 |
| Left-a | 0.040 | 0.012 to 0.069 | 0.005 | 0.014 |  | 0.010 | -0.022 to 0.042 | 0.533 | 0.878 |  | 0.050 | -0.003 to 0.103 | 0.066 | 0.128 |
| Left-b | 0.061 | 0.021 to 0.101 | 0.003 | 0.010 |  | 0.011 | -0.033 to 0.056 | 0.620 | 0.890 |  | 0.085 | 0.008 to 0.163 | 0.031 | 0.079 |
| Tip-L | -0.006 | -0.016 to 0.004 | 0.244 | 0.350 |  | -0.007 | -0.019 to 0.004 | 0.207 | 0.658 |  | -0.005 | -0.023 to 0.013 | 0.573 | 0.699 |
| Tip-a | -0.022 | -0.053 to 0.009 | 0.168 | 0.253 |  | -0.022 | -0.057 to 0.014 | 0.237 | 0.703 |  | -0.021 | -0.076 to 0.035 | 0.467 | 0.602 |
| Tip-b | -0.020 | -0.070 to 0.031 | 0.449 | 0.547 |  | -0.039 | -0.096 to 0.018 | 0.181 | 0.620 |  | -0.001 | -0.093 to 0.092 | 0.991 | 0.991 |
| **Complexion** |  |  |  |  |  |  |  |  |  |  |  |  |  |  |
| All-L | 0.138 | 0.077 to 0.198 | ＜0.001 | ＜0.001 |  | 0.048 | -0.022 to 0.117 | 0.182 | 0.600 |  | 0.206 | 0.094 to 0.318 | ＜0.001 | 0.003 |
| All-a | -0.041 | -0.142 to 0.060 | 0.427 | 0.528 |  | -0.095 | -0.209 to 0.019 | 0.102 | 0.504 |  | 0.120 | -0.062 to 0.302 | 0.195 | 0.285 |
| All-b | 0.241 | 0.158 to 0.324 | ＜0.001 | ＜0.001 |  | 0.028 | -0.078 to 0.134 | 0.607 | 0.931 |  | 0.356 | 0.219 to 0.493 | ＜0.001 | ＜0.001 |
| Forehead-L | 0.051 | 0.015 to 0.087 | 0.006 | 0.014 |  | 0.017 | -0.026 to 0.060 | 0.445 | 0.861 |  | 0.066 | 0.003 to 0.128 | 0.040 | 0.091 |
| Forehead-a | 0.062 | -0.018 to 0.142 | 0.127 | 0.195 |  | -0.016 | -0.108 to 0.076 | 0.736 | 0.885 |  | 0.150 | 0.012 to 0.289 | 0.034 | 0.082 |
| Forehead-b | 0.064 | -0.016 to 0.143 | 0.118 | 0.188 |  | -0.011 | -0.103 to 0.081 | 0.813 | 0.904 |  | 0.147 | 0.009 to 0.286 | 0.037 | 0.087 |
| Right cheek-L | 0.082 | 0.024 to 0.139 | 0.006 | 0.014 |  | 0.023 | -0.042 to 0.089 | 0.484 | 0.862 |  | 0.141 | 0.035 to 0.247 | 0.009 | 0.033 |
| Right cheek-a | -0.029 | -0.105 to 0.047 | 0.456 | 0.548 |  | -0.069 | -0.154 to 0.016 | 0.113 | 0.479 |  | 0.047 | -0.091 to 0.186 | 0.502 | 0.638 |
| Right cheek-b | 0.198 | 0.142 to 0.255 | ＜0.001 | ＜0.001 |  | 0.053 | -0.024 to 0.131 | 0.174 | 0.645 |  | 0.241 | 0.151 to 0.332 | ＜0.001 | ＜0.001 |
| Left cheek-L | 0.041 | -0.010 to 0.093 | 0.114 | 0.184 |  | -0.010 | -0.070 to 0.050 | 0.748 | 0.876 |  | 0.098 | 0.011 to 0.186 | 0.028 | 0.076 |
| Left cheek-a | -0.049 | -0.125 to 0.026 | 0.203 | 0.301 |  | -0.086 | -0.172 to -0.001 | 0.048 | 0.356 |  | 0.011 | -0.125 to 0.147 | 0.874 | 0.937 |
| Left cheek-b | 0.202 | 0.145 to 0.260 | ＜0.001 | ＜0.001 |  | 0.055 | -0.024 to 0.134 | 0.175 | 0.623 |  | 0.241 | 0148 to 0.334 | ＜0.001 | ＜0.001 |
| Periocular-L | 0.074 | 0.025 to 0.123 | 0.003 | 0.010 |  | 0.013 | -0.046 to 0.071 | 0.671 | 0.878 |  | 0.096 | 0.010 to 0.182 | 0.029 | 0.076 |
| Periocular-a | 0.048 | -0.064 to 0.159 | 0.401 | 0.503 |  | -0.003 | -0.129 to 0.123 | 0.964 | 0.964 |  | 0.130 | -0.071 to 0.330 | 0.204 | 0.293 |
| Periocular-b | 0.254 | 0.179 to 0.330 | ＜0.001 | ＜0.001 |  | 0.074 | -0.022 to 0.170 | 0.129 | 0.522 |  | 0.352 | 0.225 to 0.480 | ＜0.001 | ＜0.001 |
| Nose-L | 0.122 | 0.061 to 0.183 | ＜0.001 | 0.001 |  | 0.017 | -0.054 to 0.087 | 0.646 | 0.871 |  | 0.220 | 0.111 to 0.328 | ＜0.001 | 0.001 |
| Nose-a | -0.043 | -0.136 to 0.050 | 0.365 | 0.478 |  | -0.028 | -0.136 to 0.080 | 0.612 | 0.923 |  | 0.042 | -0.121 to 0.204 | 0.617 | 0.732 |
| Nose-b | 0.198 | 0.136 to 0.261 | ＜0.001 | ＜0.001 |  | 0.036 | -0.046 to 0.119 | 0.390 | 0.789 |  | 0.260 | 0.156 to 0.365 | ＜0.001 | ＜0.001 |
| Lip-L | 0.130 | 0.084 to 0.176 | ＜0.001 | ＜0.001 |  | 0.060 | 0.007 to 0.113 | 0.026 | 0.331 |  | 0.166 | 0.072 to 0.259 | 0.001 | 0.007 |
| Lip-a | -0.039 | -0.118 to 0.040 | 0.337 | 0.448 |  | -0.062 | -0.150 to 0.025 | 0.165 | 0.638 |  | 0.185 | 0.030 to 0.340 | 0.020 | 0.057 |
| Lip-b | 0.210 | 0.127 to 0.292 | ＜0.001 | ＜0.001 |  | 0.034 | -0.066 to 0.134 | 0.503 | 0.861 |  | 0.313 | 0.168 to 0.459 | ＜0.001 | ＜0.001 |

^a^ Other types include Special constitution, Damp-heat constitution, Phlegm dampness constitution, and Blood-stasis constitution.

The color variables (L, a, b) were defined in terms of the CIELAB space, in which L (lightness), a (red-green axis), b (Yellow-blue axis). BMI, body mass index;

CI, Confidence interval; FDR, false discovery rate.

Supplementary Table 4. Age-stratified multivariate analysis of pulse, tongue, and face diagnoses in association with spherical equivalent.

| Variables | *β* | 95% CI | *P* value |
| --- | --- | --- | --- |
|  | **Total (n=873)** | | |
| **Model 1^a^** |  |  |  |
| Age (years) | -0.319 | -0.386 to -0.251 | ＜0.001 |
| Number of myopic parents |  |  |  |
| 0 | Ref. |  |  |
| 1 | -0.697 | -0.991 to -0.403 | ＜0.001 |
| 2 | -0.787 | -1.104 to -0.470 | ＜0.001 |
| Outdoor activity time per day  on weekdays (hours) |  |  |  |
| ＜1 | Ref. |  |  |
| ≥1 to＜2 | 0.307 | 0.020 to 0.594 | 0.036 |
| ≥2 | 0.383 | 0.008 to 0.758 | 0.045 |
| Body constitution type, n (%) |  |  |  |
| Balance | Ref. |  |  |
| Qi stagnation | -0.595 | -0.922 to -0.268 | ＜0.001 |
| Yin deficiency | -0.400 | -0.779 to -0.022 | 0.038 |
| Yang deficiency | -0.594 | -1.091 to -0.097 | 0.019 |
| Qi deficiency | -0.794 | -1.417 to -0.171 | 0.012 |
| Other types^c^ | -0.177 | -0.771 to -0.417 | 0.559 |
| **Model 2^b^** |  |  |  |
| Age (years) | -0.293 | -0.363 to -0.223 | ＜0.001 |
| Number of myopic parents |  |  |  |
| 0 | Ref. |  |  |
| 1 | -0.677 | -0.971 to -0.383 | ＜0.001 |
| 2 | -0.774 | -1.091 to -0.457 | ＜0.001 |
| Body constitution type, n (%) |  |  |  |
| Balance | Ref. |  |  |
| Qi stagnation | -0.593 | -0.921 to -0.266 | ＜0.001 |
| Yin deficiency | -0.343 | -0.720 to 0.035 | 0.075 |
| Yang deficiency | -0.568 | -1.063 to -0.072 | 0.025 |
| Qi deficiency | -0.805 | -1.426 to -0.184 | 0.011 |
| Other types^c^ | -0.198 | -0.789 to 0.393 | 0.511 |
|  | **7-10 years old (n=557)*** | | |
| Age (years) | -0.530 | -0.670 to -0.390 | ＜0.001 |
| Body constitution type, n (%) |  |  |  |
| Balance | Ref. |  |  |
| Qi stagnation | -0.714 | -1.122 to -0.306 | 0.001 |
| Yin deficiency | -0.539 | -0.950 to -0.129 | 0.010 |
| Yang deficiency | -0.562 | -1.134 to 0.010 | 0.054 |
| Qi deficiency | -0.998 | -1.694 to -0.302 | 0.005 |
| Other types^c^ | -0.134 | -0.994 to 0.726 | 0.761 |
|  | **11-14 years old (n=316)** | | |
| **Model 1 ^a^** |  |  |  |
| h1 | 0.131 | 0.062 to 0.201 | ＜0.001 |
| Age (years) | -0.379 | -0.580 to -0.179 | ＜0.001 |
| Number of myopic parents |  |  |  |
| 0 | Ref. |  |  |
| 1 | -0.839 | -1.329 to -0.350 | 0.001 |
| 2 | -0.976 | -1.496 to -0.455 | ＜0.001 |
| Outdoor activity time per day  on weekdays (hours) |  |  |  |
| ＜1 | Ref. |  |  |
| ≥1 to＜2 | 0.332 | -0.130 to 0.793 | 0.159 |
| ≥2 | 0.727 | 0.056 to 1.398 | 0.034 |
| **Model 2 ^b^** |  |  |  |
| h1 | 0.132 | 0.062 to 0.202 | ＜0.001 |
| Age (years) | -0.467 | -0.664 to -0.270 | ＜0.001 |
| Number of myopic parents |  |  |  |
| 0 | Ref. |  |  |
| 1 | -0.818 | -1.311 to -0.325 | 0.001 |
| 2 | -0.945 | -1.471 to -0.418 | ＜0.001 |
| Outdoor activity time per day  on weekdays (hours) |  |  |  |
| ＜1 | Ref. |  |  |
| ≥1 to＜2 | 0.406 | -0.056 to 0.868 | 0.085 |
| ≥2 | 0.763 | 0.089 to 1.437 | 0.026 |

* For children aged 7–10 years, univariate analysis showed no significant association of pulse, tongue or face diagnosis parameters with SE.

^a^ Model 1 included the global region parameters of tongue and facial images with *P*-FDR < 0.10 in univariate analysis and VIF<5.
^b^ Model 2 included the local region parameters of tongue and facial images with *P*-FDR < 0.10 in univariate analysis and VIF<5.

^c^ Other types include Special constitution, Damp-heat constitution, Phlegm dampness constitution, and Blood-stasis constitution.

CI, Confidence interval.

Supplementary Table 5. Age-stratified univariate analysis of pulse, tongue, and face diagnoses in association with axial length.

| Variables | Total (n=873) | | | |  | 7-10 years old (n=557) | | | |  | 11-14 years old (n=316) | | | |
| --- | --- | --- | --- | --- | --- | --- | --- | --- | --- | --- | --- | --- | --- | --- |
|  | *β* | 95% CI | *P* value | *P*-FDR |  | *β* | 95% CI | *P* value | *P*-FDR |  | *β* | 95% CI | *P* value | *P*-FDR |
| Age (years) | 0.156 | 0.124 to 0.188 | ＜0.001 | ＜0.001 |  | 0.247 | 0.170 to 0.324 | ＜0.001 | ＜0.001 |  | 0.198 | 0.100 to 0.297 | ＜0.001 | 0.007 |
| BMI (kg/m^2^) | 0.050 | 0.032 to 0.069 | ＜0.001 | ＜0.001 |  | 0.024 | -0.001 to 0.049 | 0.055 | 0.445 |  | 0.049 | 0.019 to 0.080 | 0.002 | 0.045 |
| Sex |  |  |  |  |  |  |  |  |  |  |  |  |  |  |
| male | Ref. |  |  |  |  | Ref. |  |  |  |  | Ref. |  |  |  |
| female | -0.307 | -0.449 to -0.165 | ＜0.001 | ＜0.001 |  | -0.385 | -0.549 to -0.221 | ＜0.001 | ＜0.001 |  | -0.219 | -0.469 to 0.031 | 0.086 | 0.225 |
| Number of myopic parents |  |  |  |  |  |  |  |  |  |  |  |  |  |  |
| 0 | Ref. |  |  |  |  | Ref. |  |  |  |  | Ref. |  |  |  |
| 1 | 0.088 | -0.082 to 0.257 | 0.312 | 0.427 |  | 0.092 | -0.110 to 0.293 | 0.374 | 0.832 |  | 0.233 | -0.056 to 0.522 | 0.114 | 0.254 |
| 2 | 0.172 | -0.010 to 0.354 | 0.064 | 0.163 |  | 0.079 | -0.137 to 0.295 | 0.471 | 0.855 |  | 0.488 | 0.179 to 9.613 | 0.002 | 0.045 |
| Indoor near work time per day  on weekdays (hours) |  |  |  |  |  |  |  |  |  |  |  |  |  |  |
| ＜4 | Ref. |  |  |  |  | Ref. |  |  |  |  | Ref. |  |  |  |
| ≥4 to＜8 | 0.064 | -0.098 to 0.226 | 0.438 | 0.549 |  | -0.093 | -0.272 to 0.086 | 0.309 | 0.887 |  | 0.085 | -0.246 to 0.416 | 0.615 | 0.842 |
| ≥8 | 0.038 | -0.176 to 0.252 | 0.726 | 0.788 |  | -0.009 | -0.280 to 0.263 | 0.951 | 0.984 |  | -0.211 | -0.585 to 0.165 | 0.272 | 0.475 |
| Indoor near work time per day  on weekends (hours) |  |  |  |  |  |  |  |  |  |  |  |  |  |  |
| ＜4 | Ref. |  |  |  |  | Ref. |  |  |  |  | Ref. |  |  |  |
| ≥4 to＜8 | 0.284 | 0.130 to 0.437 | ＜0.001 | 0.002 |  | 0.106 | -0.069 to 0.281 | 0.235 | 0.871 |  | 0.409 | 0.109 to 0.710 | 0.007 | 0.045 |
| ≥8 | 0.305 | 0.082 to 0.528 | 0.007 | 0.027 |  | 0.054 | -0.257 to 0.365 | 0.735 | 0.991 |  | 0.292 | -0.066 to 0.649 | 0.109 | 0.255 |
| Outdoor activity time per day  on weekdays (hours) |  |  |  |  |  |  |  |  |  |  |  |  |  |  |
| ＜1 | Ref. |  |  |  |  | Ref. |  |  |  |  | Ref. |  |  |  |
| ≥1 to＜2 | -0.254 | -0.412 to -0.097 | 0.002 | 0.010 |  | -0.184 | -0.369 to 0.001 | 0.051 | 0.454 |  | -0.260 | -0.532 to 0.012 | 0.061 | 0.187 |
| ≥2  Outdoor activity time per day  on weekends (hours) | -0.292 | -0.496 to -0.088 | 0.005 | 0.022 |  | -0.133 | -0.363 to 0.098 | 0.260 | 0.890 |  | -0.428 | -0.816 to -0.041 | 0.030 | 0.103 |
| ＜1 | Ref. |  |  |  |  | Ref. |  |  |  |  | Ref. |  |  |  |
| ≥1 to＜2 | -0.251 | -0.425 to -0.078 | 0.005 | 0.022 |  | -0.194 | -0.408 to 0.020 | 0.075 | 0.477 |  | -0.225 | -0.506 to 0.057 | 0.117 | 0.254 |
| ≥2 | -0.400 | -0.579 to -0.221 | ＜0.001 | 0.000 |  | -0.240 | -0.452 to -0.028 | 0.026 | 0.386 |  | -0.422 | -0.753 to -0.091 | 0.012 | 0.049 |
| Body constitution type, n (%) |  |  |  |  |  |  |  |  |  |  |  |  |  |  |
| Balance | Ref. |  |  |  |  | Ref. |  |  |  |  | Ref. |  |  |  |
| Qi stagnation | 0.408 | 0.218 to 0.599 | ＜0.001 | ＜0.001 |  | 0.442 | 0.208 to 0.677 | ＜0.001 | 0.007 |  | 0.271 | -0.035 to 0.577 | 0.082 | 0.221 |
| Yin deficiency | 0.131 | -0.089 to 0.351 | 0.244 | 0.362 |  | 0.263 | 0.028 to 0.499 | 0.029 | 0.369 |  | 0.067 | -0.424 to 0.558 | 0.790 | 0.913 |
| Yang deficiency | 0.143 | -0.146 to 0.432 | 0.332 | 0.441 |  | 0.068 | -0.259 to 0.395 | 0.685 | 0.968 |  | 0.409 | -0.125 to 0.943 | 0.133 | 0.275 |
| Qi deficiency | 0.505 | 0.143 to 0.868 | 0.006 | 0.024 |  | 0.570 | 0.171 to 0.968 | 0.005 | 0.111 |  | 0.529 | -0.189 to 1.247 | 0.149 | 0.295 |
| Other types^a^ | 0.251 | -0.094 to 0.597 | 0.154 | 0.274 |  | 0.257 | -0.237 to 0.751 | 0.307 | 0.911 |  | 0.043 | -0.439 to 0.525 | 0.860 | 0.957 |
| **Pulse condition** |  |  |  |  |  |  |  |  |  |  |  |  |  |  |
| h1 | 0.007 | -0.011 to 0.025 | 0.465 | 0.575 |  | -0.006 | -0.030 to 0.018 | 0.643 | 0.987 |  | -0.045 | -0.077 to -0.013 | 0.006 | 0.053 |
| h3 | 0.018 | -0.009 to 0.045 | 0.192 | 0.311 |  | 0.006 | -0.025 to 0.163 | 0.686 | 0.954 |  | -0.004 | -0.053 to 0.044 | 0.866 | 0.952 |
| h4 | -0.004 | -0.041 to 0.033 | 0.830 | 0.890 |  | 0.019 | -0.023 to 0.061 | 0.368 | 0.840 |  | -0.095 | -0.161 to -0.028 | 0.005 | 0.056 |
| h5 | 0.006 | -0.143 to 0.155 | 0.936 | 0.980 |  | -0.113 | -0.330 to 0.105 | 0.309 | 0.887 |  | -0.313 | -0.542 to -0.084 | 0.007 | 0.045 |
| t4 | 0.306 | -0.938 to 1.550 | 0.630 | 0.692 |  | -0.010 | -1.431 to 1.411 | 0.989 | 0.989 |  | 1.424 | -0.820 to 3.669 | 0.214 | 0.414 |
| t5 | 0.527 | -0.146 to 1.199 | 0.125 | 0.247 |  | 0.092 | -0.646 to 0.829 | 0.808 | 1.000 |  | 0.806 | -0.556 to 2.169 | 0.246 | 0.447 |
| t | 0.547 | -0.002 to 1.097 | 0.051 | 0.142 |  | 0.190 | -0.426 to 0.806 | 0.546 | 0.917 |  | 0.817 | -0.227 to 1.861 | 0.125 | 0.265 |
| h3/h1 | -0.222 | -0.593 to 0.150 | 0.242 | 0.365 |  | -0.043 | -0.476 to 0.389 | 0.844 | 1.000 |  | 0.542 | -0.184 to 1.268 | 0.144 | 0.291 |
| h4/h1 | -1.003 | -1.478 to -0.528 | ＜0.001 | 0.000 |  | -0.152 | -0.750 to 0.445 | 0.618 | 0.965 |  | -1.326 | -2.127 to -0.525 | 0.001 | 0.045 |
| h5/h1 | -1.125 | -2.906 to 0.656 | 0.216 | 0.337 |  | -0.890 | -2.915 to 1.135 | 0.389 | 0.844 |  | -3.784 | -7.868 to 0.299 | 0.069 | 0.192 |
| As | 0.004 | 0.001 to 0.008 | 0.014 | 0.048 |  | 0.004 | -0.001 to 0.008 | 0.055 | 0.445 |  | -0.004 | -0.010 to 0.003 | 0.266 | 0.473 |
| Ad | 0.006 | 0.001 to 0.011 | 0.022 | 0.073 |  | 0.003 | -0.003 to 0.009 | 0.323 | 0.846 |  | 0.001 | -0.007 to 0.009 | 0.853 | 0.961 |
| **Tongue color** |  |  |  |  |  |  |  |  |  |  |  |  |  |  |
| All-L | -0.020 | -0.043 to 0.004 | 0.096 | 0.214 |  | 0.010 | -0.018 to 0.037 | 0.495 | 0.881 |  | -0.056 | -0.096 to -0.016 | 0.006 | 0.053 |
| All-a | 0.011 | -0.026 to 0.047 | 0.570 | 0.659 |  | 0.016 | -0.026 to 0.059 | 0.449 | 0.850 |  | 0.005 | -0.059 to 0.068 | 0.886 | 0.950 |
| All-b | -0.036 | -0.079 to 0.008 | 0.108 | 0.229 |  | 0.022 | -0.033 to 0.076 | 0.438 | 0.866 |  | -0.028 | -0.101 to 0.044 | 0.440 | 0.699 |
| Middle-L | -0.016 | -0.037 to 0.004 | 0.108 | 0.229 |  | 0.012 | -0.011 to 0.036 | 0.300 | 0.954 |  | -0.045 | -0.080 to -0.010 | 0.012 | 0.049 |
| Middle-a | 0.008 | -0.019 to 0.035 | 0.572 | 0.653 |  | 0.003 | -0.028 to 0.034 | 0.848 | 0.993 |  | 0.011 | -0.037 to 0.058 | 0.664 | 0.882 |
| Middle-b | -0.020 | -0.058 to 0.018 | 0.293 | 0.414 |  | 0.020 | -0.027 to 0.066 | 0.409 | 0.827 |  | -0.009 | -0.073 to 0.055 | 0.778 | 0.911 |
| Root-L | 0.002 | -0.014 to 0.020 | 0.987 | 0.998 |  | 0.008 | -0.008 to 0.025 | 0.322 | 0.868 |  | -0.012 | -0.036 to 0.011 | 0.310 | 0.531 |
| Root-a | 0.008 | -0.021 to 0.037 | 0.595 | 0.670 |  | 0.013 | -0.022 to 0.048 | 0.468 | 0.868 |  | 0.004 | -0.046 to 0.053 | 0.885 | 0.961 |
| Root-b | -0.010 | -0.046 to 0.027 | 0.612 | 0.681 |  | 0.020 | -0.025 to 0.065 | 0.392 | 0.831 |  | 0.002 | -0.059 to 0.063 | 0.946 | 0.968 |
| Right-L | -0.022 | -0.045 to 0.001 | 0.067 | 0.166 |  | 0.003 | -0.024 to 0.031 | 0.818 | 1.000 |  | -0.052 | -0.091 to -0.013 | 0.009 | 0.050 |
| Right-a | 0.020 | -0.016 to 0.057 | 0.276 | 0.396 |  | 0.029 | -0.014 to 0.072 | 0.183 | 0.776 |  | 0.011 | -0.052 to 0.074 | 0.732 | 0.892 |
| Right-b | -0.038 | -0.081 to 0.005 | 0.080 | 0.192 |  | 0.021 | -0.033 to 0.076 | 0.439 | 0.849 |  | -0.032 | -0.103 to 0.039 | 0.377 | 0.610 |
| Left-L | -0.016 | -0.038 to 0.006 | 0.147 | 0.273 |  | 0.007 | -0.019 to 0.033 | 0.599 | 0.987 |  | -0.039 | -0.076 to -0.002 | 0.041 | 0.135 |
| Left-a | 0.019 | -0.017 to 0.055 | 0.296 | 0.412 |  | 0.022 | -0.020 to 0.064 | 0.304 | 0.933 |  | 0.021 | -0.043 to 0.084 | 0.519 | 0.745 |
| Left-b | -0.040 | -0.082 to 0.002 | 0.061 | 0.160 |  | 0.012 | -0.041 to 0.066 | 0.647 | 0.976 |  | -0.028 | -0.098 to 0.043 | 0.440 | 0.699 |
| Tip-L | -0.024 | -0.047 to -0.001 | 0.044 | 0.126 |  | 0.009 | -0.018 to 0.037 | 0.507 | 0.885 |  | -0.052 | -0.091 to -0.013 | 0.009 | 0.050 |
| Tip-a | -0.013 | -0.039 to 0.013 | 0.337 | 0.441 |  | -0.008 | -0.038 to 0.022 | 0.601 | 0.973 |  | -0.014 | -0.060 to 0.032 | 0.560 | 0.791 |
| Tip-b | -0.029 | -0.070 to 0.011 | 0.156 | 0.272 |  | 0.024 | -0.026 to 0.073 | 0.347 | 0.835 |  | -0.041 | -0.109 to 0.028 | 0.243 | 0.451 |
| **Tongue shape** |  |  |  |  |  |  |  |  |  |  |  |  |  |  |
| Medium | Ref. |  |  |  |  | Ref. |  |  |  |  | Ref. |  |  |  |
| Swollen | -0.009 | -0.172 to 0.154 | 0.915 | 0.969 |  | 0.034 | -0.157 to 0.225 | 0.727 | 0.995 |  | -0.099 | -0.380 to 0.181 | 0.487 | 0.722 |
| Thin | -0.152 | -0.377 to 0.074 | 0.187 | 0.308 |  | -0.124 | -0.380 to 0.133 | 0.345 | 0.853 |  | -0.149 | -0.559 to 0.260 | 0.475 | 0.729 |
| **Fur color** |  |  |  |  |  |  |  |  |  |  |  |  |  |  |
| All-L | -0.002 | -0.007 to 0.003 | 0.372 | 0.473 |  | 0.001 | -0.005 to 0.008 | 0.916 | 0.971 |  | 0.001 | -0.009 to 0.010 | 0.914 | 0.957 |
| All-a | -0.006 | -0.023 to 0.011 | 0.496 | 0.605 |  | 0.002 | -0.018 to 0.021 | 0.862 | 0.971 |  | 0.007 | -0.026 to 0.039 | 0.692 | 0.880 |
| All-b | -0.012 | -0.036 to 0.011 | 0.313 | 0.422 |  | 0.003 | -0.024 to 0.029 | 0.854 | 0.974 |  | 0.002 | -0.044 to 0.048 | 0.931 | 0.963 |
| Middle-L | 0.001 | -0.004 to 0.006 | 0.946 | 0.979 |  | 0.001 | -0.004 to 0.005 | 0.799 | 1.000 |  | 0.004 | -0.004 to 0.011 | 0.338 | 0.568 |
| Middle-a | 0.004 | -0.009 to 0.017 | 0.558 | 0.662 |  | 0.001 | -0.014 to 0.016 | 0.906 | 0.995 |  | 0.019 | -0.005 to 0.043 | 0.113 | 0.258 |
| Middle-b | 0.001 | -0.019 to 0.025 | 0.963 | 0.985 |  | 0.005 | -0.016 to 0.026 | 0.648 | 0.961 |  | 0.017 | -0.021 to 0.055 | 0.374 | 0.616 |
| Root-L | -0.002 | -0.006 to 0.002 | 0.249 | 0.363 |  | 0.001 | -0.005 to 0.006 | 0.956 | 0.978 |  | -0.002 | -0.009 to 0.004 | 0.492 | 0.718 |
| Root-a | -0.009 | -0.023 to 0.005 | 0.210 | 0.334 |  | 0.001 | -0.016 to 0.018 | 0.907 | 0.984 |  | -0.009 | -0.034 to 0.016 | 0.481 | 0.726 |
| Root-b | -0.008 | -0.026 to 0.009 | 0.339 | 0.437 |  | 0.003 | -0.018 to 0.023 | 0.789 | 1.000 |  | -0.005 | -0.035 to 0.026 | 0.759 | 0.913 |
| Right-L | -0.004 | -0.008 to 0.001 | 0.109 | 0.220 |  | -0.001 | -0.006 to 0.005 | 0.834 | 1.000 |  | -0.002 | -0.011 to 0.007 | 0.677 | 0.873 |
| Right-a | -0.012 | -0.027 to 0.004 | 0.133 | 0.257 |  | -0.001 | -0.018 to 0.016 | 0.932 | 0.976 |  | -0.004 | -0.033 to 0.025 | 0.770 | 0.914 |
| Right-b | -0.019 | -0.040 to 0.003 | 0.085 | 0.194 |  | -0.002 | -0.027 to 0.022 | 0.848 | 0.993 |  | -0.009 | -0.051 to 0.033 | 0.668 | 0.874 |
| Left-L | -0.003 | -0.008 to 0.001 | 0.182 | 0.306 |  | -0.001 | -0.006 to 0.005 | 0.844 | 1.000 |  | -0.001 | -0.010 to 0.009 | 0.966 | 0.977 |
| Left-a | -0.009 | -0.024 to 0.006 | 0.224 | 0.344 |  | -0.001 | -0.018 to 0.016 | 0.878 | 0.977 |  | 0.002 | -0.026 to 0.030 | 0.886 | 0.950 |
| Left-b | -0.016 | -0.037 to 0.006 | 0.149 | 0.271 |  | -0.003 | -0.027 to 0.022 | 0.832 | 1.000 |  | -0.001 | -0.042 to 0.040 | 0.973 | 0.973 |
| Tip-L | 0.006 | 0.001 to 0.011 | 0.036 | 0.110 |  | 0.005 | -0.001 to 0.011 | 0.114 | 0.564 |  | 0.008 | -0.001 to 0.018 | 0.095 | 0.235 |
| Tip-a | 0.021 | 0.005 to 0.038 | 0.011 | 0.039 |  | 0.017 | -0.002 to 0.036 | 0.085 | 0.473 |  | 0.029 | -0.001 to 0.058 | 0.055 | 0.175 |
| Tip-b | 0.026 | 0.001 to 0.053 | 0.054 | 0.146 |  | 0.029 | -0.002 to 0.060 | 0.064 | 0.438 |  | 0.031 | -0.018 to 0.080 | 0.216 | 0.409 |
| **Complexion** |  |  |  |  |  |  |  |  |  |  |  |  |  |  |
| All-L | -0.072 | -0.104 to -0.040 | ＜0.001 | 0.000 |  | -0.029 | -0.067 to 0.009 | 0.130 | 0.579 |  | -0.103 | -0.162 to -0.044 | 0.001 | 0.045 |
| All-a | 0.048 | -0.006 to 0.101 | 0.081 | 0.190 |  | 0.067 | 0.006 to 0.129 | 0.032 | 0.356 |  | -0.017 | -0.113 to 0.079 | 0.729 | 0.901 |
| All-b | -0.080 | -0.124 to -0.035 | ＜0.001 | 0.001 |  | 0.006 | -0.051 to 0.064 | 0.824 | 1.000 |  | -0.104 | -0.178 to -0.030 | 0.006 | 0.053 |
| Forehead-L | -0.031 | -0.050 to -0.011 | 0.002 | 0.010 |  | -0.011 | -0.034 to 0.012 | 0.342 | 0.870 |  | -0.043 | -0.076 to -0.010 | 0.011 | 0.047 |
| Forehead-a | -0.013 | -0.055 to 0.030 | 0.560 | 0.656 |  | 0.030 | -0.019 to 0.080 | 0.233 | 0.902 |  | -0.064 | -0.137 to 0.010 | 0.089 | 0.226 |
| Forehead-b | -0.013 | -0.056 to 0.029 | 0.546 | 0.657 |  | 0.028 | -0.022 to 0.078 | 0.270 | 0.890 |  | -0.062 | -0.135 to 0.012 | 0.099 | 0.238 |
| Right cheek-L | -0.041 | -0.071 to -0.010 | 0.010 | 0.037 |  | -0.012 | -0.047 to 0.024 | 0.514 | 0.880 |  | -0.071 | -0.127 to -0.015 | 0.013 | 0.048 |
| Right cheek-a | 0.028 | -0.013 to 0.068 | 0.178 | 0.305 |  | 0.038 | -0.008 to 0.084 | 0.104 | 0.544 |  | 0.009 | -0.064 to 0.082 | 0.816 | 0.931 |
| Right cheek-b | -0.069 | -0.099 to -0.039 | ＜0.001 | 0.000 |  | -0.010 | -0.051 to 0.032 | 0.649 | 0.947 |  | -0.069 | -0.118 to -0.019 | 0.006 | 0.053 |
| Left cheek-L | -0.023 | -0.050 to 0.005 | 0.104 | 0.226 |  | -0.002 | -0.034 to 0.030 | 0.912 | 0.978 |  | -0.044 | -0.090 to 0.003 | 0.065 | 0.187 |
| Left cheek-a | 0.031 | -0.009 to 0.071 | 0.133 | 0.257 |  | 0.041 | -0.005 to 0.087 | 0.083 | 0.492 |  | 0.017 | -0.055 to 0.088 | 0.645 | 0.870 |
| Left cheek-b | -0.074 | -0.105 to -0.043 | ＜0.001 | 0.000 |  | -0.018 | -0.061 to 0.024 | 0.400 | 0.828 |  | -0.066 | -0.117 to -0.016 | 0.010 | 0.045 |
| Periocular-L | -0.054 | -0.080 to -0.028 | ＜0.001 | 0.000 |  | -0.025 | -0.056 to 0.006 | 0.117 | 0.548 |  | -0.064 | -0.110 to -0.019 | 0.005 | 0.056 |
| Periocular-a | 0.001 | -0.059 to 0.070 | 0.990 | 0.990 |  | 0.014 | -0.053 to 0.082 | 0.678 | 0.973 |  | -0.019 | -0.125 to 0.087 | 0.724 | 0.908 |
| Periocular-b | -0.091 | -0.132 to -0.050 | ＜0.001 | 0.000 |  | -0.030 | -0.081 to 0.022 | 0.259 | 0.922 |  | -0.093 | -0.162 to -0.023 | 0.009 | 0.050 |
| Nose-L | -0.061 | -0.093 to -0.028 | ＜0.001 | 0.001 |  | -0.018 | -0.056 to 0.020 | 0.352 | 0.824 |  | -0.092 | -0.150 to -0.034 | 0.002 | 0.045 |
| Nose-a | 0.054 | 0.004 to 0.103 | 0.033 | 0.105 |  | 0.040 | -0.019 to 0.098 | 0.183 | 0.776 |  | 0.025 | -0.061 to 0.111 | 0.564 | 0.784 |
| Nose-b | -0.071 | -0.105 to -0.038 | ＜0.001 | 0.000 |  | -0.001 | -0.047 to 0.044 | 0.988 | 0.999 |  | -0.083 | -0.140 to -0.026 | 0.004 | 0.051 |
| Lip-L | -0.060 | -0.084 to -0.035 | ＜0.001 | 0.000 |  | -0.029 | -0.058 to -0.001 | 0.045 | 0.445 |  | -0.067 | -0.116 to -0.017 | 0.009 | 0.050 |
| Lip-a | 0.044 | 0.002 to 0.086 | 0.040 | 0.119 |  | 0.062 | 0.015 to 0.109 | 0.010 | 0.178 |  | -0.078 | -0.160 to 0.004 | 0.063 | 0.187 |
| Lip-b | -0.080 | -0.124 to -0.036 | ＜0.001 | 0.001 |  | -0.014 | -0.068 to 0.040 | 0.611 | 0.971 |  | -0.092 | -0.170 to -0.014 | 0.021 | 0.075 |

^a^ Other types include Special constitution, Damp-heat constitution, Phlegm dampness constitution, and Blood-stasis constitution.

The color variables (L, a, b) were defined in terms of the CIELAB space, in which L (lightness), a (red-green axis), b (Yellow-blue axis). BMI, body mass index; CI, Confidence interval; FDR, false discovery rate.

Supplementary Table 6. Age-stratified multivariate analysis of pulse, tongue, and face diagnoses in association with axial length.

| Variables | *β* | 95% CI | *P* value |
| --- | --- | --- | --- |
|  | **Total (n=873)** | | |
| **Model 1^a^** |  |  |  |
| Age (years) | 0.122 | 0.081 to 0.162 | ＜0.001 |
| Sex |  |  |  |
| male | Ref. |  |  |
| female | -0.348 | -0.487 to -0.208 | ＜0.001 |
| Outdoor activity time per day  on weekends (hours) |  |  |  |
| ＜1 | Ref. |  |  |
| ≥1 to＜2 | -0.206 | -0.376 to -0.037 | 0.017 |
| ≥2 | -0.176 | -0.361 to 0.009 | 0.063 |
| Body constitution type, n (%) |  |  |  |
| Balance | Ref. |  |  |
| Qi stagnation | 0.365 | 0.186 to 0.544 | ＜0.001 |
| Yin deficiency | 0.204 | -0.003 to 0.411 | 0.053 |
| Yang deficiency | 0.267 | -0.006 to 0.540 | 0.055 |
| Qi deficiency | 0.518 | 0.178 to 0.858 | 0.003 |
| Other types^c^ | 0.109 | -0.214 to 0.433 | 0.508 |
| **Model 2 ^b^** |  |  |  |
| Age (years) | 0.118 | 0.079 to 0.158 | ＜0.001 |
| Sex |  |  |  |
| male | Ref. |  |  |
| female | -0.342 | -0.480 to -0.203 | ＜0.001 |
| Outdoor activity time per day  on weekends (hours) |  |  |  |
| ＜1 | Ref. |  |  |
| ≥1 to＜2 | -0.188 | -0.356 to -0.020 | 0.028 |
| ≥2 | -0.177 | -0.362 to 0.007 | 0.059 |
| Body constitution type, n (%) |  |  |  |
| Balance | Ref. |  |  |
| Qi stagnation | 0.366 | 0.187 to 0.545 | ＜0.001 |
| Yin deficiency | 0.197 | -0.009 to 0.403 | 0.061 |
| Yang deficiency | 0.284 | 0.010 to 0.557 | 0.042 |
| Qi deficiency | 0.493 | 0.153 to 0.832 | 0.004 |
| Other types^c^ | 0.085 | -0.238 to 0.408 | 0.605 |
|  | **7-10 years old (n=557)*** | | |
| Age (years) | 0.263 | 0.187 to 0.339 | ＜0.001 |
| Sex |  |  |  |
| male | Ref. |  |  |
| female | -0.382 | -0.541 to -0.223 | ＜0.001 |
| Body constitution type, n (%) |  |  |  |
| Balance | Ref. |  |  |
| Qi stagnation | 0.459 | 0.238 to 0.680 | ＜0.001 |
| Yin deficiency | 0.264 | 0.041 to 0.486 | 0.020 |
| Yang deficiency | 0.223 | -0.087 to 0.533 | 0.159 |
| Qi deficiency | 0.659 | 0.282 to 1.036 | 0.001 |
| Other types^c^ | 0.203 | -0.263 to 0.668 | 0.394 |
|  | **11-14 years old (n=316)** | | |
| **Model 1 ^a^** |  |  |  |
| h1 | -0.083 | -0.130 to -0.037 | ＜0.001 |
| h5 | -0.413 | -0.629 to -0.197 | ＜0.001 |
| Age (years) | 0.188 | 0.075 to 0.301 | 0.001 |
| BMI (kg/m^2^) | 0.037 | 0.009 to 0.066 | 0.011 |
| Number of myopic parents |  |  |  |
| 0 | Ref. |  |  |
| 1 | 0.218 | -0.045 to 0.480 | 0.104 |
| 2 | 0.418 | 0.139 to 0.697 | 0.003 |
| **Model 2 ^b^** |  |  |  |
| h1 | -0.085 | -0.132 to -0.038 | ＜0.001 |
| h5 | -0.425 | -0.642 to -0.208 | ＜0.001 |
| Age (years) | 0.184 | 0.077 to 0.292 | 0.001 |
| BMI (kg/m^2^) | 0.042 | 0.013 to 0.072 | 0.005 |
| Number of myopic parents |  |  |  |
| 0 | Ref. |  |  |
| 1 | 0.228 | -0.033 to 0.490 | 0.087 |
| 2 | 0.449 | 0.169 to 0.728 | 0.002 |

* For children aged 7–10 years, univariate analysis showed no significant association of pulse, tongue or face diagnosis parameters with SE.

^a^ Model 1 included the global region parameters of tongue and facial images with *P*-FDR < 0.10 in univariate analysis and VIF<5.

^b^ Model 2 included the local region parameters of tongue and facial images with *P*-FDR < 0.10 in univariate analysis and VIF<5.

^c^ Other types include Special constitution, Damp-heat constitution, Phlegm dampness constitution, and Blood-stasis constitution.

BMI, body mass index; CI, Confidence interval.

Supplementary Table 7. Correlations of pulse wave amplitudes with the scores of five major TCM body constitutions.

| Variables | Groups | Balance  (n=446, 51.09%) | |  | Qi stagnation  (n=171, 19.59%) | |  | Yin deficiency  (n=117, 13.40%) | |  | Yang deficiency  (n=61, 6.99%) | |  | Qi deficiency  (n=37, 4.24%) | |
| --- | --- | --- | --- | --- | --- | --- | --- | --- | --- | --- | --- | --- | --- | --- | --- |
|  |  | *r* | *P* |  | *r* | *P* |  | *r* | *P* |  | *r* | *P* |  | *r* | *P* |
| h1 (mm) | Total (n=873) | 0.081 | 0.086 |  | -0.189 | **0.013** |  | -0.144 | 0.123 |  | -0.239 | 0..063 |  | -0.419 | **0.010** |
|  | Myopia (n=464) | 0.080 | 0.248 |  | -0.202 | **0.036** |  | -0.110 | 0.357 |  | -0.348 | **0.035** |  | -0.476 | **0.034** |
|  | Non-myopia (n=409) | 0.069 | 0.287 |  | -0.156 | 0.222 |  | -0.173 | 0.256 |  | -0.053 | 0.804 |  | -0.193 | 0.457 |
| h3 (mm) | Total (n=873) | 0.089 | 0.060 |  | -0.175 | **0.022** |  | 0.029 | 0.760 |  | -0.223 | 0.084 |  | -0.301 | 0.071 |
|  | Myopia (n=464) | 0.109 | 0.121 |  | -0.162 | 0.095 |  | 0.130 | 0.278 |  | -0.421 | **0.040** |  | -0.317 | 0.174 |
|  | Non-myopia (n=409) | 0.067 | 0.301 |  | -0.158 | 0.216 |  | -0.176 | 0.248 |  | -0.061 | 0.722 |  | -0.062 | 0.812 |
| h4 (mm) | Total (n=873) | 0.106 | **0.026** |  | -0.052 | 0.500 |  | -0.028 | 0.761 |  | -0.375 | **0.003** |  | -0.405 | **0.013** |
|  | Myopia (n=464) | 0.097 | 0.170 |  | -0.074 | 0.449 |  | -0.018 | 0.880 |  | -0.344 | **0.037** |  | -0.482 | **0.031** |
|  | Non-myopia (n=409) | 0.107 | 0.097 |  | -0.029 | 0.820 |  | -0.017 | 0.906 |  | -0.309 | 0.052 |  | -0.084 | 0.749 |
| h5 (mm) | Total (n=873) | 0.054 | 0.258 |  | 0.031 | 0.690 |  | -0.170 | 0.067 |  | -0.146 | 0.263 |  | -0.108 | 0.524 |
|  | Myopia (n=464) | 0.015 | 0.833 |  | 0.030 | 0.749 |  | -0.177 | 0.138 |  | -0.251 | 0.134 |  | -0.225 | 0.341 |
|  | Non-myopia (n=409) | 0.083 | 0.199 |  | -0.009 | 0.943 |  | -0.075 | 0.625 |  | 0.099 | 0.644 |  | -0.120 | 0.648 |

Due to the low proportion of participants with Special Constitution (n=22, 2.52%), Damp‑heat (n=8, 0.92%), Phlegm dampness (n=6, 0.69%), and Blood‑stasis constitutions (n=5, 0.57%), correlation analyses with pulse wave amplitudes were not performed for these constitution types.
